# Evolutionary transitions between beneficial and phytopathogenic *Rhodococcus* challenge disease management

**DOI:** 10.7554/eLife.30925

**Published:** 2017-12-12

**Authors:** Elizabeth A Savory, Skylar L Fuller, Alexandra J Weisberg, William J Thomas, Michael I Gordon, Danielle M Stevens, Allison L Creason, Michael S Belcher, Maryna Serdani, Michele S Wiseman, Niklaus J Grünwald, Melodie L Putnam, Jeff H Chang

**Affiliations:** 1 Department of Botany and Plant PathologyOregon State UniversityCorvallisUnited States; 2 Molecular and Cellular Biology ProgramOregon State UniversityCorvallisUnited States; 3 Horticultural Crops Research LaboratoryUnited States Department of Agriculture and Agricultural Research ServiceCorvallisUnited States; 4 Center for Genome ResearchOregon State UniversityCorvallisUnited States; University of ChicagoUnited States

**Keywords:** mutualism, virulence, evolutionary transition, horizontal gene transfer, Rhodococcus, plant growth promoting bacteria, Other

## Abstract

Understanding how bacteria affect plant health is crucial for developing sustainable crop production systems. We coupled ecological sampling and genome sequencing to characterize the population genetic history of *Rhodococcus* and the distribution patterns of virulence plasmids in isolates from nurseries. Analysis of chromosome sequences shows that plants host multiple lineages of *Rhodococcus*, and suggested that these bacteria are transmitted due to independent introductions, reservoir populations, and point source outbreaks. We demonstrate that isolates lacking virulence genes promote beneficial plant growth, and that the acquisition of a virulence plasmid is sufficient to transition beneficial symbionts to phytopathogens. This evolutionary transition, along with the distribution patterns of plasmids, reveals the impact of horizontal gene transfer in rapidly generating new pathogenic lineages and provides an alternative explanation for pathogen transmission patterns. Results also uncovered a misdiagnosed epidemic that implicated beneficial *Rhodococcus* bacteria as pathogens of pistachio. The misdiagnosis perpetuated the unnecessary removal of trees and exacerbated economic losses.

## eLife digest

All organisms live in a world teeming with bacteria. Some bacteria are beneficial and, for example, provide their hosts with nutrients. Others cause harm, for example, by stealing nutrients and causing disease. Many bacteria can also gain DNA from other bacteria, and the genes encoded within the new DNA can help them to live with other organisms. This can start the bacteria on an evolutionary path to becoming beneficial or harmful.

*Rhodococcus* are bacteria that live in association with many species of plants, including trees. Most are harmless but some cause disease. Plants infected with harmful *Rhodococcus* can show deformed growth, which causes major losses to the nursery industry.

Savory, Fuller, Weisberg et al. set out to understand how disease-causing *Rhodococcus* are introduced into nurseries, if they are transferred between nurseries, whether they persist in nurseries, and how to limit their spread. It turns out that harmless *Rhodococcus* are beneficial to plants. However, if these harmless bacteria gain a certain DNA molecule – called a virulence plasmid – they can convert into harmful bacteria. Further analysis showed that some nurseries repeatedly acquired the harmful bacteria. The pattern of affected nurseries suggested that some might have purchased diseased plants from a common provider. In other cases, the sources remained a mystery.

Savory et al. also report that, contrary to previous findings, there is no evidence to support the diagnosis that *Rhodococcus* without a virulence plasmid are responsible for an unusual growth problem that has plagued the pistachio industry. In recent years, this incorrect diagnosis led to trees being unnecessarily destroyed, worsening the economic losses.

These findings suggest that genes moving between bacteria can dramatically change how those bacteria interact with the organisms in which they live. It needs to be shown whether this is an exceptional process, unique to only certain groups of bacteria, or if it is more widespread in nature. These findings could inform future disease management strategies to better protect agricultural systems.

## Introduction

Symbioses are persistent and intimate interactions between organisms. In pathogenic interactions, one partner benefits at the expense of the other. In mutualistic symbioses, specific partners interact and reciprocally benefit. Associative symbioses are a variation of mutualism in which there is lower specificity between interacting partners (Drogue et al., 2012). In agricultural systems, practices are employed to limit pathogens, to introduce nitrogen-fixing mutualistic rhizobia and to restore associative symbionts such as plant growth-promoting bacteria (PGPB). PGPB can directly promote the growth of plants and protect against pathogens (Barea et al., 2005; Pieterse et al., 2014).

The beneficial or parasitic outcomes of symbioses, especially those involving environmentally acquired partners, are often not guaranteed. The health of the host, location of the symbiont on the host, or unregulated proliferation of the symbiont can lead to alternative outcomes (Lin and Koskella, 2015). The genotype of the symbiont is also a critical factor, as horizontal gene transfer (HGT) can lead to the acquisition of new genes that innovate genomes, driving evolutionary transitions and establishing new lineages of beneficial or pathogenic symbionts (Soucy et al., 2015). In some pathogenic symbionts, however, HGT does not bestow the genome with innovative functions, nor do these genomes exhibit substantive changes. Rather, the few horizontally acquired genes encode products that reprogram core genes, thereby co-opting the genome for virulence (Letek et al., 2010).

*Rhodococcus* is a genus of Gram-positive bacteria with members that persist in a variety of terrestrial and aquatic ecosystems (de Carvalho et al., 2014; Larkin et al., 2005). *Rhodococcus* includes taxonomic groups with members that have been repeatedly recovered from leaf and root tissues of various species of plants (Bai et al., 2015; Bodenhausen et al., 2013; Bulgarelli et al., 2012; Hong et al., 2015, 2016; Lebeis et al., 2015; Lundberg et al., 2012; Qin et al., 2009, 2011; Salam et al., 2017). It has been suggested that hosts enrich for members of *Rhodococcus* because of the beneficial traits of the bacteria (Hong et al., 2015, 2016).

Plant-associated *Rhodococcus* species are better known as pathogens (Putnam and Miller, 2007). Two clades of *Rhodococcus* include members that can cause disease in over 100 genera of plants (Creason et al., 2014a2014a; Putnam and Miller, 2007). Herbaceous plants are the most commonly affected whereas woody plants are less frequently infected. Disease symptoms include leafy galls, witches’-brooms, and other disfiguring growths. Pathogenic isolates of *Rhodococcus*, typified by the most-studied isolate D188, require three virulence loci that are most frequently found clustered on virulence plasmids (Crespi et al., 1992; Stes et al., 2011). These plasmids are approximately 200 kb in length and are linear replicons (Francis et al., 2012; Creason et al., 2014b). Some of the *fas* (*fasciation*) genes are necessary for disease and encode proteins that synthesize and modify cytokinins, which are predicted to be secreted effectors (Crespi et al., 1994; Pertry et al., 2009). The *fasR* gene, predicted to be a transcriptional regulator, is also necessary for pathogenicity (Temmerman et al., 2000). *att* (*attenuation*) mutants are reportedly attenuated in disease and thus implicated in virulence (Crespi et al., 1992; Maes et al., 2001). The *vicA* gene, which encodes malate synthase, an enzyme in the glyoxylate cycle, is the only locus encoded on the chromosome implicated in virulence (Vereecke et al., 2002).

Pathogenic *Rhodococcus* are particularly problematic in agricultural settings that produce plants for their aesthetic value. A variety of biotic and abiotic stresses, some induced by anthropogenic practices, cause symptoms that are confused with those caused by *Rhodococcus* (Putnam and Miller, 2007). Furthermore, isolates of *Rhodococcus* that lack virulence genes are often cultured from symptomatic tissues (Creason et al., 2014b; Nikolaeva et al., 2009). Multiple tests are used to confirm that a plant is infected by pathogenic *Rhodococcus*. Bacteria must exhibit the proper morphology on selective media and be taxonomically assigned to *Rhodococcus*. The bacteria must have virulence genes and must cause disease symptoms in susceptible indicator plants. Because the *fasR* gene and some of the *fas* genes are necessary for pathogenicity of the bacteria, their detection is sufficient to confirm pathogenicity of *Rhodococcus* isolates.

In 2011, populations of micropropagated pistachio UCB-1 (*Pistacia atlantica* × *Pistacia integerrima*) rootstocks planted in commercial fields began showing an unusual phenotype (Stamler et al., 2015a, 2015b). Aerial phenotypes of ‘pistachio bushy top syndrome’ include shortened internodes, loss of apical dominance, stem galls, and reduced grafting success. Estimates suggest that more than 1 million trees grown on 25,000–30,000 acres were affected. *Rhodococcus* isolates were cultured from symptomatic plants, and when inoculated onto UCB-1, they caused morphological changes to the hosts (Stamler et al., 2015b). This was the first report of pistachio being susceptible to *Rhodococcus*. Subsequent release of the genome sequences for PBTS1 and PBTS2, the reported outbreak strains, indicated they lack virulence loci (Stamler et al., 2016). The detection of *vicA* was used as evidence for pathogenic *Rhodococcus* bacteria and to guide management practices, the most extreme and costly being the removal of entire orchards. A second incidence of pistachio bushy top syndrome occurred in 2016, resulting in the destruction of 1.5 million nursery trees.

We determined and analyzed genome sequences from over 80 isolates of *Rhodococcus*, mostly collected from symptomatic herbaceous plants grown in production settings. Analysis of chromosomal sequences shows that plants host multiple lineages of *Rhodococcus*. Isolates that lack virulence plasmids can promote changes to the architecture of roots, but if a virulence plasmid is acquired, the isolates transition to being pathogenic. The analysis of chromosomal sequences of pathogens revealed the potential for multiple infections and reservoir populations at nursery sites, as well as for point source outbreaks. However, the distribution patterns of virulence plasmids suggested that agricultural systems can be locations that promote evolutionary transitions and the rapid generation of new lineages of pathogens, providing an alternative route for the spread of pathogens. Last, our results challenge previous conclusions that *Rhodococcus* isolates lacking virulence genes are causative agents of pistachio bushy top syndrome, suggesting that the pistachio syndrome was likely misdiagnosed (Stamler et al., 2015a, 2015b).

## Results

### Epidemiological links in nurseries

We used a genomic epidemiological approach to study the transmission patterns of *Rhodococcus* in a plant agricultural system. Sixty isolates were collected hierarchically across space and time. Multiple isolates were cultured from the same symptomatic tissues or from different plants grown at the same production site (Supplementary file 1A). Previously sequenced isolates, many also collected from production sites, were included (Supplementary file 1A; Creason et al., 2014b). The nursery sources of the isolates were anonymized.

**Figure 1.**
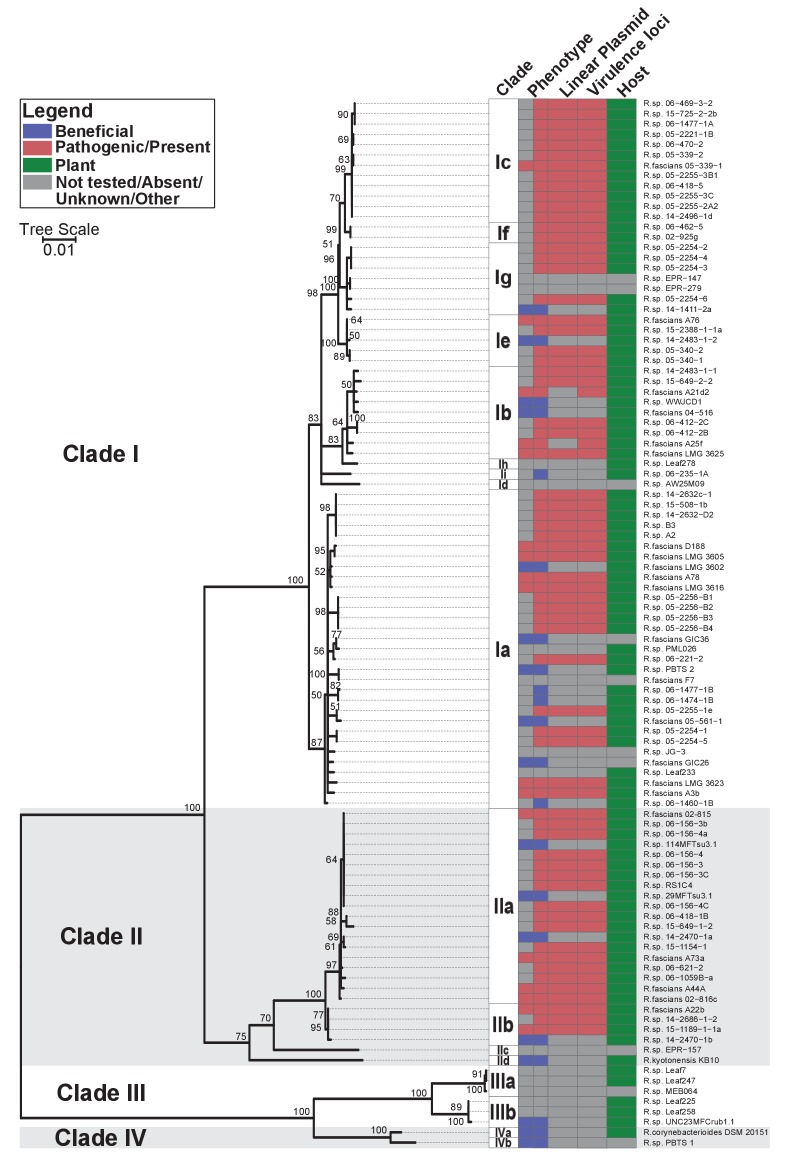
Plant-associated isolates of *Rhodococcus* form four sister clades. Multi-locus sequence analysis maximum likelihood tree of plant-associated isolates of *Rhodococcus*. Translated sequences for *ftsY*, *infB*, *rpoB*, *rsmA*, *secY*, *tsaD*, and *ychF* from 104 members of *Rhodococcus* were identified using TBLASTN, aligned, and used to generate a multi-locus maximum likelihood tree. Clade designations are based on analysis of average nucleotide identity (Supplementary file 1B). Columns indicate the features of the corresponding isolate. Grey bars indicate not tested, absent, unknown, or other for phenotype, linear plasmid, virulence loci, and host columns, respectively. The left-half of the column corresponding to phenotype indicates a confirmed phenotype, whereas the right-half indicates an inference based on the presence or absence of virulence genes.

Phylogenetic analysis placed the 60 isolates within *Rhodococcus* Clades I and II, which also included the 15 previously confirmed pathogenic isolates (Figure 1; Creason et al., 2014b). The two clades are sisters to Clades III and IV. Clade III consists of isolates that were cultured from microbiota of the model plant*Arabidopsis thaliana* (Bai et al., 2015; Bodenhausen et al., 2013; Bulgarelli et al., 2012; Lebeis et al., 2015; Lundberg et al., 2012). No member of Clades III or IV have virulence genes. The four clades are distinct, and separated by a long branch, from the other species of *Rhodococcus* (Figure 1—figure supplement 1). The isolates in the four clades were operationally classified into 17 species, indicated with a lowercase letter, of which 14 have at least one isolate cultured from a plant (Supplementary file 1B).

**Figure 1—figure supplement 1.**
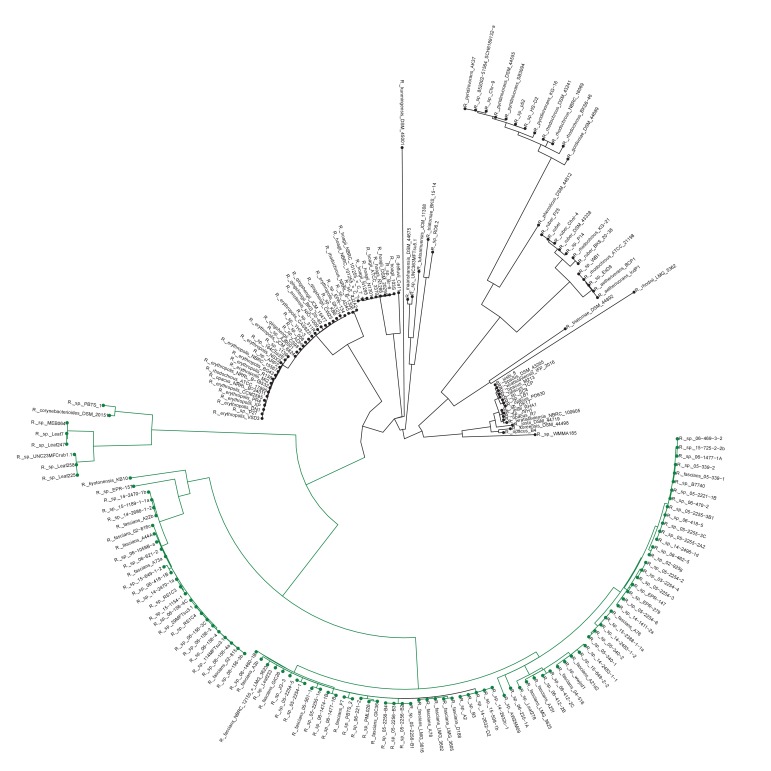
Plant-associated *Rhodococcus* form a distinct clade. Multi-locus sequence analysis maximum likelihood tree of isolates of the *Rhodococcus* genus. Translated sequences for *ftsY*, *infB*, *rpoB*, *rsmA*, *secY*, *tsaD*, and *ychF* from 199 members of *Rhodococcus* were identified using TBLASTN, aligned, and used to generate a multi-locus maximum likelihood tree.

Fifty-one of the newly sequenced isolates encode virulence genes. We inspected their genome assemblies as well as those from previously sequenced pathogenic isolates (Creason et al., 2014b). All but four of the 66 genome assemblies had *att*, *fasR*, and *fas* on the same contig as pFi_009. The *fas* locus is present in a region that is conserved in pFiD188, the virulence plasmid of isolate D188, suggested to be necessary for plasmid replication and maintenance (Francis et al., 2012). The pFi_009 gene is predicted to encode a telomere-associated protein hypothesized to be necessary for the replication of the linear virulence plasmid. The genome assemblies of isolates 06-469-3-2 and 05-2254-6 had contig breaks that disrupted the linkages between virulence and plasmid-associated loci, but these contigs were nonetheless similar in composition and are co-linear to the reference plasmid sequence. The average sequencing coverage of plasmids relative to that of corresponding chromosomes was 1.89 ± 0.56. Only three assemblies had coverages less than 1.0 but all three had virulence loci on the same contig as pFi_009. A21d2 and A25f, previously sequenced, are exceptional because the virulence loci are encoded in their chromosomes (Creason et al., 2014b). Therefore, of the 66 isolates confirmed or inferred to be pathogenic, 64 carry a virulence plasmid. Pathogenic isolates were assigned to eight different species (Figure 1).

**Figure 2.**
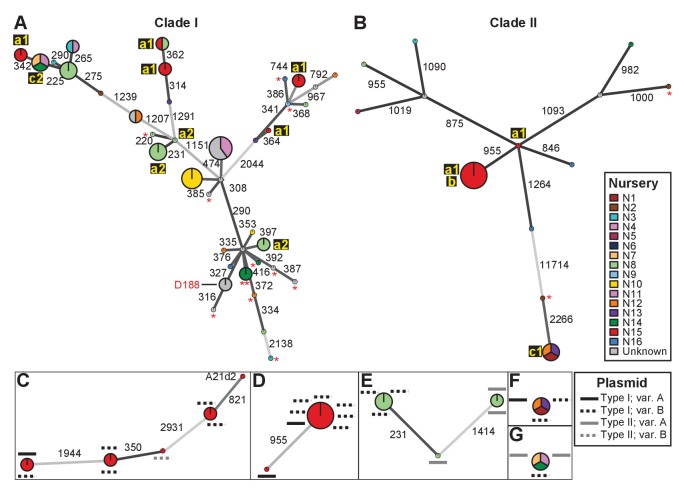
Analysis of SNPs reveals three transmission patterns of pathogenic *Rhodococcus*. Minimum spanning networks of isolates of (**A**) Clade I and (**B**) Clade II. Each genotype is displayed as a circle, with sizes scaled to represent the number of associated isolates (smallest = 1 isolate). Colors represent the source of the isolates (see key), with coloring proportional to the ratio of isolates from each source. Lower-case letters and numbers (a1, a2, b, c1, and c2) highlight potential transmission patterns; see panels **C–G**). Asterisks = lacking virulence genes. The genotype that includes D188 is indicated. (**C, D**) Minimum spanning networks of pathogenic isolates belonging to Clade I (**C;** ‘a1’) and Clade II (**D;** ‘a1’ and ‘b’) from nursery N15. A21d2 lacks a virulence plasmid and its virulence loci are present in the chromosome. (**E**) The minimum spanning network for isolates of pathogenic isolates from nursery N8 (‘a2’). (**F**) The epidemiological link ‘c1’ between isolates from nurseries N1, N12, and N13. (**G**) The epidemiological link ‘c2’ between isolates from nurseries N7, N11, and N14. Plasmid types and their variants are mapped onto each of the nodes (see key). Numbers adjacent to connecting lines indicate the number of SNPs that separate each genotype. The lengths of connecting lines are arbitrary; gray lines indicate distances that exceed an arbitrary threshold.

We identified single nucleotide polymorphisms (SNPs) for 82 isolates and used these SNPs to define genotypes and to assemble two clade-specific minimum spanning networks (Supplementary files 1C and 1D; Figure 2). The genotypes show pairwise differences in between 220 and 11,714 SNPs. We mapped nursery information onto the network, which provided information on potential transmission patterns (Figure 2). In this figure, the ‘a’ identification is associated with nurseries in which we identified evidence of multiple and independent infections. Plants from nursery N15, indicated with ‘a1’, were infected by five genotypes belonging to Clade I and two belonging to Clade II. Nursery N8 (‘a2’) and several others were also associated with multiple genotypes. However, nursery N8 had a single host plant that was infected by at least three genotypes that represented six of the cultured isolates. The three isolates within one of these genotypes differ by up to 20 pairwise SNPs, whereas the two isolates in one of the other genotypes have no differences (Supplementary file 1C). The third genotype is separated from the other two by 231 and 1414 pairwise SNPs (Figure 2E). Epidemiological link ‘b’ was detected in nursery N15. The corresponding genotype includes seven isolates from Clade IIa, sampled four years apart from *Campanula* plants (Supplementary file 1A). These isolates are separated by 0–2 pairwise SNPs. Epidemiological links designated as ‘c’ were also made between isolates collected from geographically separated nurseries. Nurseries N1, N12, and N13 (‘c1’) had isolates of the associated genotype (Clade IIb) that are separated by 1–4 pairwise SNPs and were collected up to 13 years apart from *Leucanthemum* and *Geranium* plants. Nurseries N7, N11, and N14 (‘c2’) had isolates of the associated genotype (Clade Ia) that were separated by 12–20 pairwise SNPs. The isolates were collected nine years apart, and from *Veronica* plants.

### Multiple distribution patterns of plasmids occur in isolates cultured from agricultural settings

**Figure 3.**
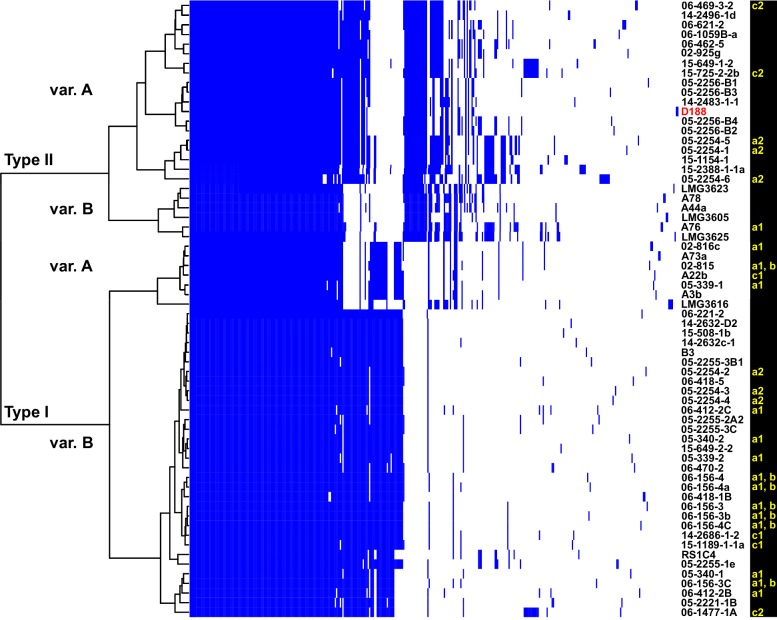
Analysis of plasmid variation reveals multiple patterns in distribution. Rows indicate genes present in (blue) or absent from (white) the plasmids of isolates (listed to the right; D188 is labeled in red for reference). Columns represent individual genes. Type categories were determined on the basis of phylogenetic analysis of the core genes. INDEL variants, delineated by gray and white shading, were determined on the basis of the cladogram. The plasmid carried by LMG3616 is enclosed by dotted lines. The lower-case letters and numbers (a1, a2, b, c1, and c2) listed along the right, relate isolates and their plasmids to the potential transmission patterns indicated in Figure 2.

The virulence plasmids of 64 plant pathogenic *Rhodococcus* isolates were categorized on the basis of the phylogenetic analysis of 123 genes that are present in at least 95% of the virulence plasmids, and sub-categorized on the basis of patterns of gene presence/absence (Figure 3; Figure 3—figure supplement 1; Supplementary file 1E). The phylogeny has strong support for two major plasmid types, as well as one unique type only carried by isolate LMG3616 (Figure 3—figure supplement 1). The plasmid sequences within the major clades are conserved and provided few informative nucleotide polymorphisms for sub-categorizing plasmids. The greatest contribution to plasmid diversity is gene gain and loss, and the relative clustering of plasmids based on the presence/absence of genes allowed subgrouping into INDEL variants, as indicated with uppercase letters in Figure 3. At the level of plasmid type, presence/absence categorization was identical to the phylogeny (Figure 3—figure supplement 1). The genes that define each INDEL variant are often present in largely contiguous regions in the plasmid, and could have been acquired as blocks (Francis et al., 2012).

**Figure 3—figure supplement 1.**
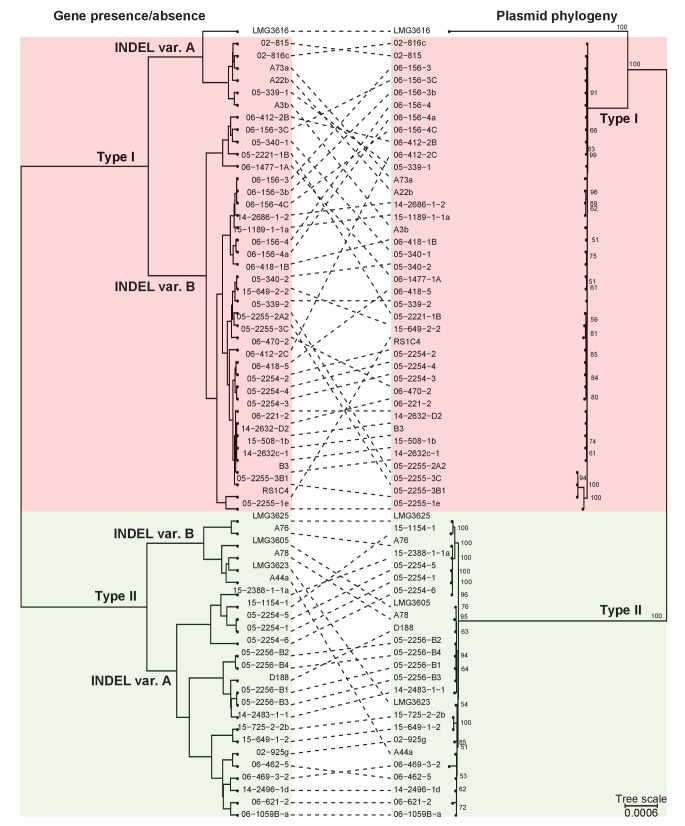
Presence/absence-cladogram and phylogeny support the classification scheme for the virulence plasmid. The dashed lines show congruency in results from methods used to cluster plasmid types; (left) plasmids were clustered in a cladogram on the basis of the presence/absence of genes and (right) in a phylogenetic tree on the basis of the concatenated sequences of 123 genes that are present in more than 95% of the virulence plasmid sequences. Red and green blocks delineate Type I and Type II plasmids, respectively.

**Figure 3—figure supplement 2.**
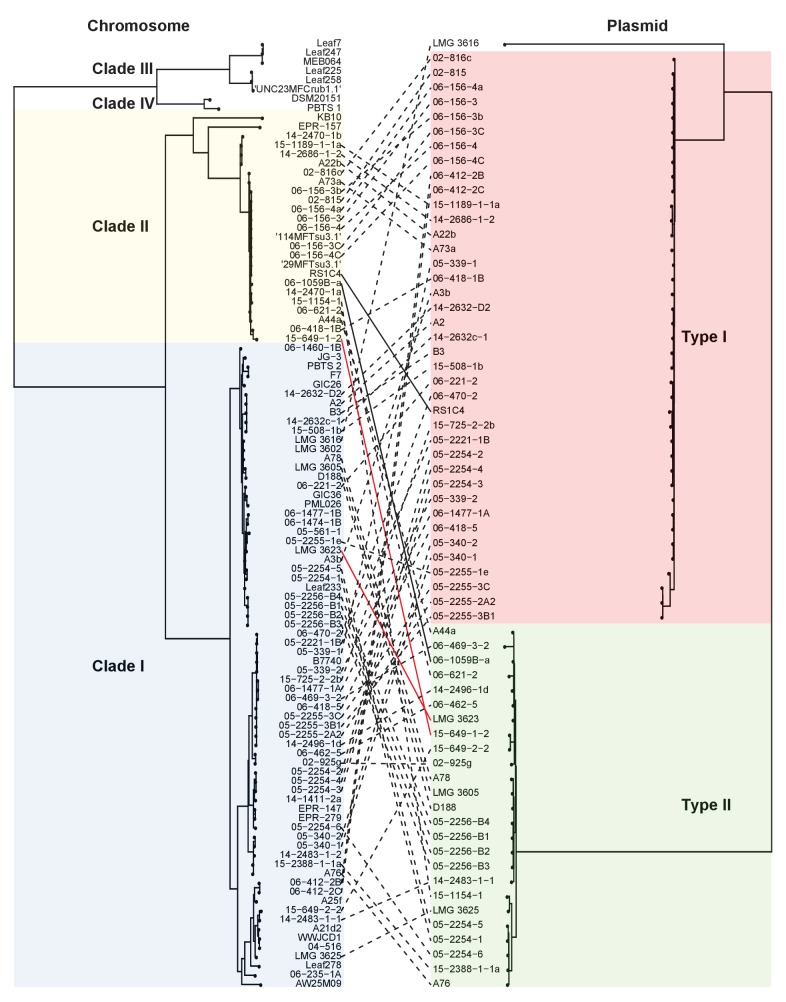
Both types of virulence plasmids are present in both clades of pathogenic *Rhodococcus*. The multilocus sequence analysis (MLSA) phylogenetic tree of the four plant-associated clades was associated with the phylogenetic tree on the basis of the concatenated sequences of 123 genes that are present in more than 95% of the virulence plasmid sequences. The lines between trees show relationships between isolates and plasmids. The two pairs of solid (black and red) lines highlight examples of closely related isolates that have divergent plasmid types and distantly- related isolates that have similar plasmid types.

We next characterized the distribution of plasmids in epidemiologically linked isolates and showed that the patterns of plasmids were inconsistent with those expected of transmission between isolates of a chromosomal genotype (Figures 2C–G and 3). One genotype associated with ‘a1’ (the most left node of network; Figure 2C) includes isolates 05-339-1 and 05-339-2, which differ by seven pairwise SNPs and which carry Type IA and Type IB plasmids, respectively. The genotype associated with ‘b’ includes isolate 02–815, which carries a Type IA plasmid (Figure 2D). This isolate is epidemiologically linked to six isolates (0–2 pairwise SNPs), but the isolates collected four years later carry a Type IB virulence plasmid. Likewise, the genotype associated with ‘c1’ includes isolate A22b, which carries a Type IA virulence plasmid and is linked to two isolates carrying Type IB plasmids (Figure 2F). The most striking deviation is found in the genotype associated with ‘c2’ (Figure 2G). Isolate 06-1477-1A carries a Type IB plasmid and is epidemiologically linked to two isolates that carry a Type IIA plasmid. Even the two Type IIA plasmids are dissimilar as one has a block of genes that, along with other gene INDELs, distinguishes it from the other.

The diverse genotypes of *Rhodococcus* isolated from nurseries can carry similar plasmid types and these types are not taxonomically restricted. Isolates 06-412-2B of Clade Ib, 05-340-1 of Clade Ie and 06-156-3c of Clade IIa, all of group ‘a1’ detected in nursery N15, have Type IB plasmids that differ by only a few gene INDELs (Figure 2C and D). The phylogeny shows that there are cases in which isolates from the same clade, such as RS1C4 and 06-1059B-a (solid black lines), carry different types of plasmids (Figure 3—figure supplement 2; solid black lines). It was also observed that distantly related *Rhodococcus* isolates carry the same type of plasmid. For example, 15-649-1-2 of Clade II and LMG 3623 of Clade I both carry Type II plasmids (Figure 3—figure supplement 2; solid red lines).

Two pathogenic isolates were excluded from the analysis because the virulence loci are present in their chromosomes (Creason et al., 2014b). A25f was recovered from nursery N12, whereas A21d2 was recovered from nursery N15 (Supplementary file 1A).

### *Rhodococcus* isolates that lack the virulence gene promote changes in root architecture

In this dataset, we identified nine isolates of *Rhodococcus *that lack virulence genes. We also identified five such isolates in a previous study, and often fail to detect virulence genes while diagnosing *Rhodococcus* cultured from diseased plants (Creason et al., 2014b). Others have implied that these are strains that have lost the plasmid (Nikolaeva et al., 2009). The genetic diversity of co-existing isolates described here suggests otherwise (Figure 1; Supplementary files 1A-D). Pathogenic 06-1477-1A and virulence-gene-lacking 06-1477-1B were isolated from a symptomatic *Veronica* plant, belong to Clades Id and Ia, respectively, and have 1521 pairwise SNPs. Pathogenic isolate 14-2483-1-1 (Clade Ib) was cultured from the same symptomatic plant as virulence-gene-lacking isolate 14-2483-1-2 (Clade Ie), and the two differ by 2820 SNPs. Interestingly, 14-2483-1-2 has the *attR* and *attX* virulence genes, as well as 228 nucleotides of the *attA* virulence gene, on a contig that is dissimilar in sequence and greater in length than the virulence plasmids. The first two coding sequences and the intergenic regions have ≥88% nucleotide identity to corresponding sequences in D188. For *attA*, only the first 65 nucleotides are identical to its homolog in D188. Results from PCR confirmed that the structure of the locus was not a result of misassembly. Two virulence-gene-lacking isolates, 14-2470-1a and 14-2470-1b, were cultured from a symptomatic plant. These two isolates are in Clades IIa and IIb, respectively, and differ by 13,858 SNPs. Of the isolates cultured and tested from this plant, no pathogenic isolate was detected.

An alternative explanation for the presence of genetically diverse, virulence-gene-lacking isolates of *Rhodococcus* is that such isolates are beneficial and enriched for by plants. We therefore compared the symbiosis phenotype of seven virulence-plasmid-lacking isolates that represent the four clades against four virulence-gene-carrying isolates, D188, A44a, A25f, and A21d2 (Supplementary file 1F; Creason et al., 2014b; Desomer et al., 1988; Lundberg et al., 2012; Miteva et al., 2004). The virulence-gene-carrying isolates were previously determined to be pathogenic, and were selected on the basis of having variations in the structure and sequence of their virulence loci (Creason et al., 2014b). PBTS1 and PBTS2, implicated as outbreak strains and cultured from the leaf endophytic compartment of pistachio, were also included (Stamler et al., 2015b).

**Figure 4.**
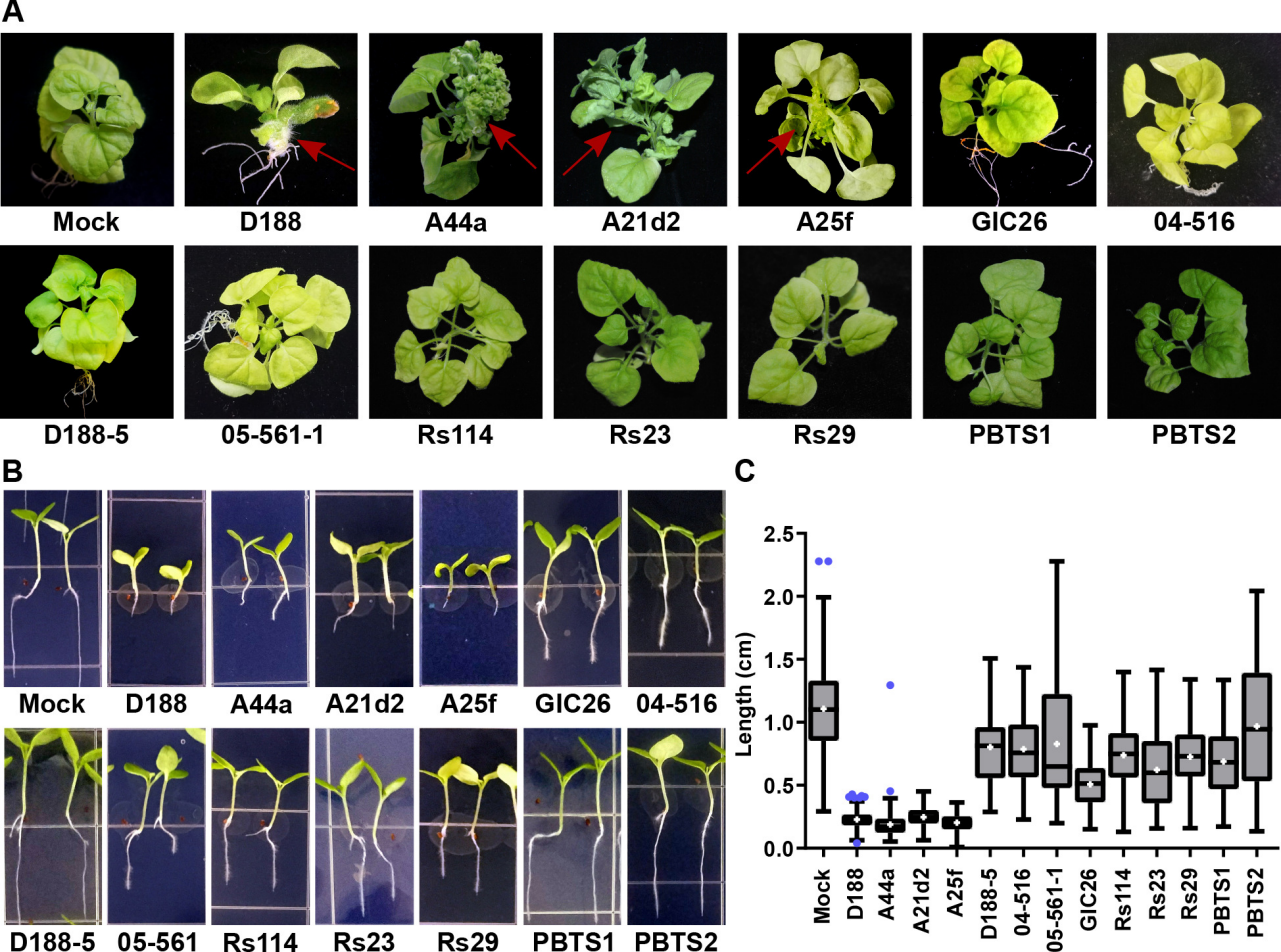
The *att, fasR,* and *fas* loci are necessary for the pathogenicity of *Rhodococcus.* (**A**) Representative images of leafy galls on *N. benthamiana*. Isolates of *Rhodococcus* were inoculated at the apical meristem. Red arrows indicate the leafy galls. Rs114, Rs23, and Rs29 are abbreviations for isolates 114MFTsu3.1, UNC23MFCrub1.1, and 29MFTsu3.1, respectively. (**B**) Representative images of the root length of seedlings. Three-day-old *N. benthamiana* seedlings were inoculated with theindicated isolate of *Rhodococcus* or water (mock) and grown vertically for seven days under constant light. Isolates D188, A44a, A21d2, and A25f are the only isolates with virulence genes .(**C**) Quantification of seedling root length. All treatments, except for PBTS2, were significant compared to the mock treatment. 10.7554/eLife.30925.010Figure 4—source data 1.Lengths of *N. benthamiana *seedling roots 7 days after inoculation with wild type *Rhodococcus*isolates.

The seven virulence-gene-lacking isolates, as well as PBTS1 and PBTS2, failed to cause disease when inoculated onto the meristems of mature *Nicotiana benthamiana*. The four pathogenic isolates caused leafy galls (Figure 4A). These four were also the only ones tested that caused significant inhibition of root elongation, thickening of the stem, and terminal arrest at the cotyledon stage (no primary growth or development of lateral roots), when assayed on seedlings of *N. benthamiana* (Figure 4B and C). We examined plants up to two months after inoculation, and seedlings remained terminally arrested. Most isolates reduced the vertical growth of roots, compared to that of mock-inoculated plants, but inhibition by virulence-gene-lacking isolates was more variable and not as severe as that measured in the roots of pathogen-inoculated seedlings (p-values were <0.0001 except for the treatment with PBTS2 [p-value = 0.1153]). Importantly, seedlings inoculated with virulence-gene-lacking isolates did not show thickening of the stem or terminal arrest at the cotyledon stage, morphological changes associated with disease (Figure 4).

**Figure 5.**
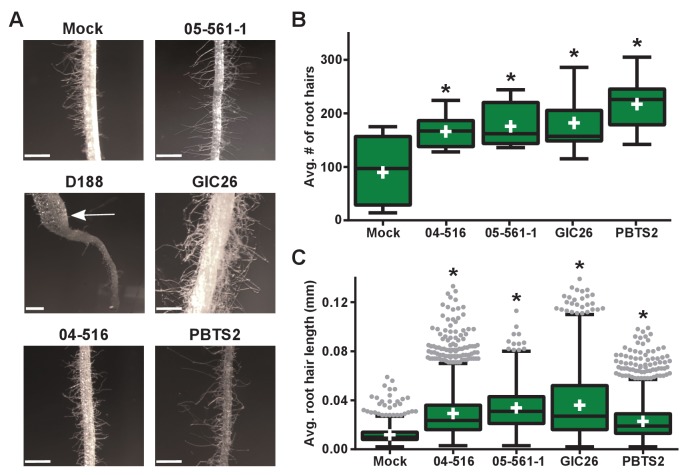
Plant-associated *Rhodococcus* bacteria cause changes to the root architecture of seedlings. (**A**) Representative images of root hairs of *N. benthamiana* that were inoculated with isolates of *Rhodococcus*. Images were taken 25 days post inoculation (dpi). The white arrow indicates the thicker stem induced only by isolate D188. Scale bars are 0.5 mm. (**B**) Quantification of average root hair number at 25 dpi. All root hairs were manually counted for at least five seedlings per treatment. (**C**) Quantification of root hair lengths at 25 dpi. All root hairs were manually measured for at least five seedlings per treatment. For **B** and **C**, data were repeated in two independent biological replicates. * indicates a significant difference compared to the mock treatment. 10.7554/eLife.30925.012Figure 5—source data 1.Numbers of *N. benthamiana *seedling root hairs 25 days after inoculation with wild type *Rhodococcus *isolates. 10.7554/eLife.30925.013Figure 5—source data 2.Lengths of *N. benthamianaroot* hairs 25 days after inoculation with wild type *Rhodococcus *isolates.

Instead, we noticed that all virulence-gene-lacking isolates caused changes to the architecture of the roots (Figure 4B). Relative to mock-inoculated plants, there were proliferations in root hairs and the plants had more lateral roots or earlier development of lateral roots. The former change was quantified in seedlings that were inoculated with members from a subset of the virulence-gene-lacking isolates. There were significant increases in the number of root hairs (averages ranging from 166.1 to 217.3; p-values were all ≤0.0045), compared to mock-inoculated plants (average of 89.8; Figure 5A and B). The isolates varied in their effect, with PBTS2 having the strongest measurable effect (217.3; p-value<0.0001). GIC26 provoked the most visually striking proliferation of root hairs and its extreme effect challenged our ability to count and measure root hairs accurately (Figure 5A; p-value=0.0003). The average length of the root hairs was significantly longer following inoculation with the four isolates of *Rhodococcus* (averages ranged from 0.02286 to 0.03604 mm vs 0.01186 mm in mock-inoculated plants; p-values were all <0.0001; Figure 5C). Pathogenic isolate D188 was included as a control, but the root hairs of plants that were inoculated with this isolate were too sparse in number to warrant quantification (Figure 5A). Five additional isolates, including 14-2483-1-2 which has part of the *att* locus, were tested and shown to cause changes to the architecture of the roots of plants (Figure 5—figure supplement 1).

**Figure 5—figure supplement 1.**
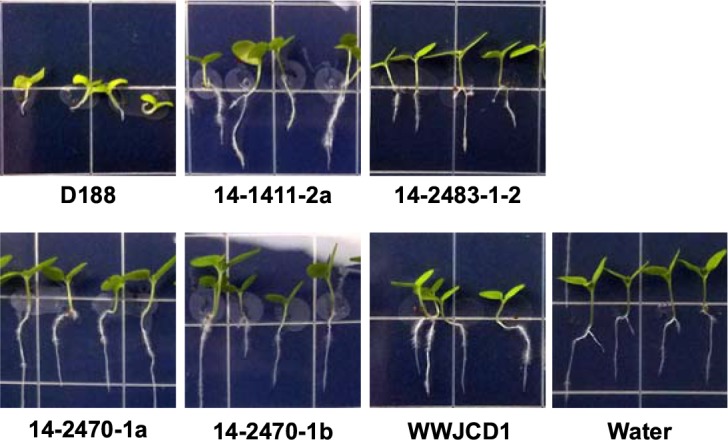
Five additional virulence-gene-lacking isolates of *Rhodococcus* cause changes to root architecture. Representative images of seedlings inoculated with the indicated isolates of *Rhodococcus* or water (mock). The root lengths of seedlings inoculated with isolates lacking virulence genes were different from those of seedlings inoculated with isolate D188.

**Figure 5—figure supplement 2.**
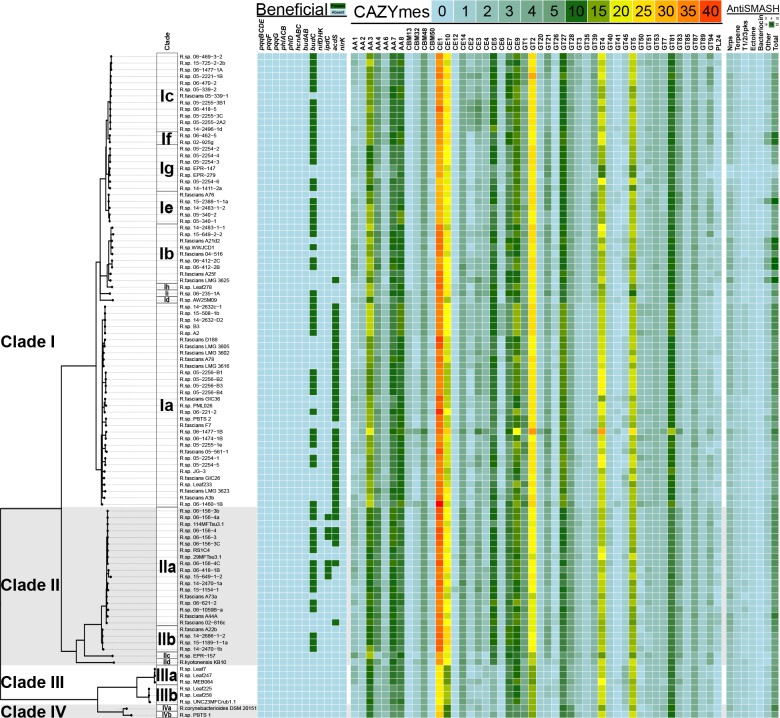
Heatmap of genes and functions enriched in clades of plant-associated *Rhodococcus*. Genes associated with plant-beneficial functions were used as queries in TBLASTN searches for homologs in *Rhodococcus* (green = presence [>40% identity, >70% length]; cyan = absence). For the CAZYmes, only the 48 most highly represented classes are shown. The key shows the number of homologs detected. Putative secondary metabolite biosynthetic clusters were identified using antiSMASH. The key shows the number of loci predicted for each category.

We analyzed the genome sequences to identify genes that are potentially involved in providing beneficial traits (Figure 5—figure supplement 2; Bruto et al., 2014; Glick, 2012; Sparacino-Watkins et al., 2014). Few homologs or pathways were associated with beneficial traits or an endophytic lifestyle. There was also no obvious correlation with symbiosis phenotype. Some isolates have a homolog predicted to encode 1-aminocyclopropane-1-carboxylate (ACC) deaminase, but this sequence had a strong phylogenetic signal, and is predominantly in members of Clade Ia (Glick, 2014). No complete tryptophan-dependent auxin biosynthetic pathway was identified (Spaepen et al., 2007). The most highly represented classes of carbohydrate active enzymes (CAZYmes) are members of carbohydrate esterase groups CE1 and CE10. CE1 includes xylanases, which degrade hemicellulose (Lombard et al., 2014). CE10 consists of cholinoesterases, a group of enzymes that act on non-carbohydrate sources. In addition, we identified cutinases (C5) and pectate lyases (PL22). These classes of enzymes may contribute to the endophytic lifestyle of *Rhodococcus*. There are 108 genes that are enriched in the genomes of isolates from the four plant-associated clades (Supplementary file 1G). Forty genes are annotated as hypothetical and many others lack sufficient information in their annotations. AntiSMASH analysis identified anywhere from 1 to ~ 20 loci that may be involved in the production of secondary metabolites (Figure 5—figure supplement 2; Blin et al., 2017). Few had sufficient similarities to previously characterized loci to allow the inference of the identity of the metabolite. Whether and how the functions of these genes contribute to the plant-associated lifestyle are unknown.

### The virulence plasmid is sufficient to transition *Rhodococcus* from being potentially beneficial to being pathogenic

**Figure 6.**
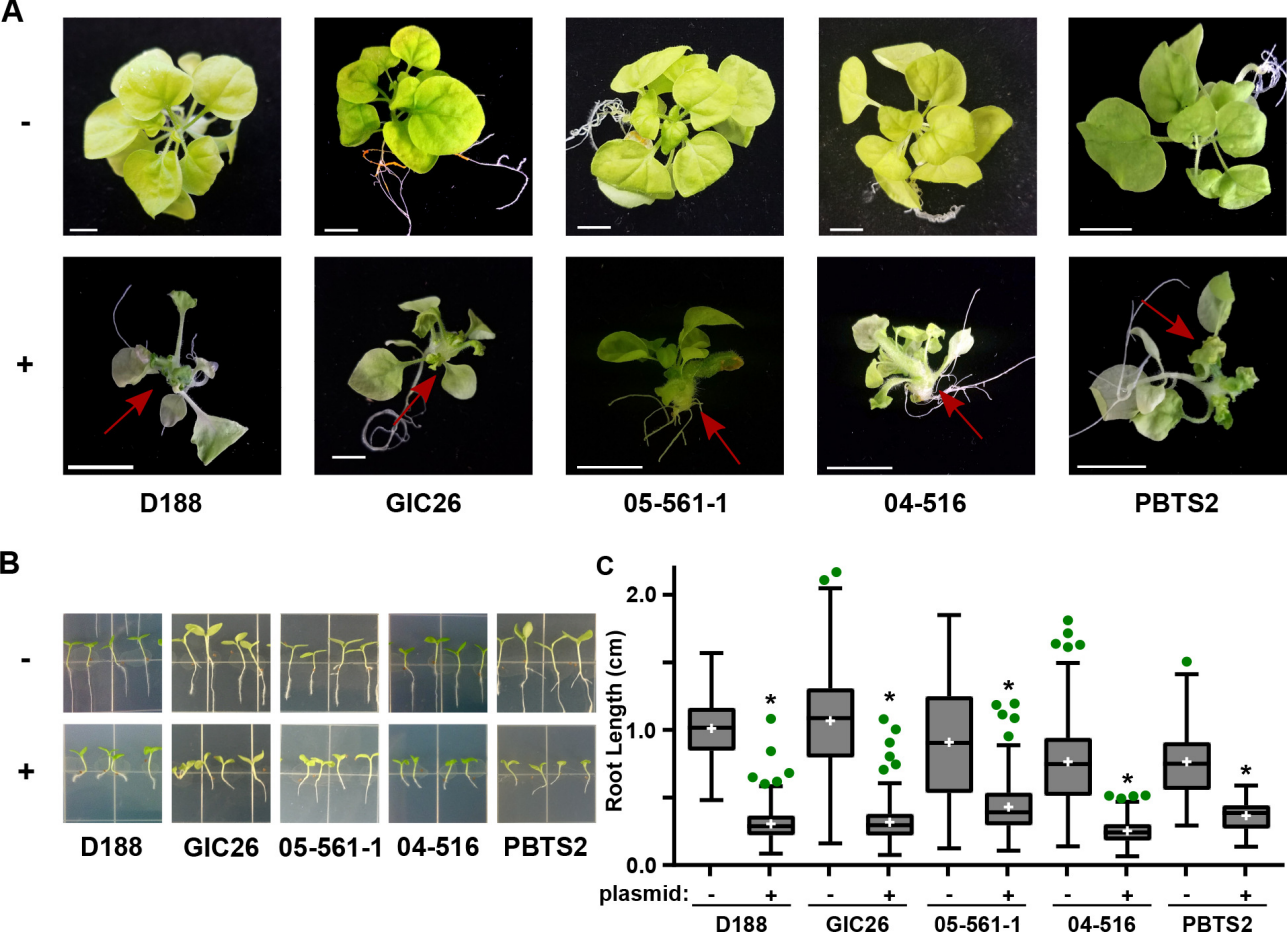
Plasmid pFiD188 with functional *fasR and fas* is sufficient to transition *Rhodococcus* isolates to phytopathogens. (**A**) Representative images of leafy galls on *N. benthamiana*. Red arrows indicate leafy galls. Images for GIC26, 04–516, and PBTS2 are repeated from Figure 1. (**B**) Representative images of the root lengths of seedlings. Three-day-old *N. benthamiana* seedlings were inoculated with the indicated isolate of *Rhodococcus* or water (mock) and grown vertically for seven days under constant light. (**C**) Quantification of the root lengths of *N. benthamiana* seedlings. In all panels, -/+ indicates absence or presence of pFiD188Δ*att*. * indicates a significant difference compared to plants treated with the corresponding genotype lacking the plasmid. 10.7554/eLife.30925.022Figure 6—source data 1.Lengths of *N. benthamiana *seedling roots 7 days after inoculation with *Rhodococcus*isolates +/- pFiD188Δ*att.*

The pFiD188Δ*att* virulence plasmid was successfully conjugated into a subset of the *Rhodococcus* isolates that originally lacked virulence genes. This plasmid variant encodes *fasR* and *fasA-F*, but has a kanamycin resistance gene that disrupts *attR*, *attX*, and *attA-G* (Maes et al., 2001). Regardless, plasmid pFiD188Δ*att* is sufficient for isolate D188 to cause disease in mature plants and seedlings, and plants that were treated with a strain containing this plasmid were no different from those infected with D188 (p-value>0.9999; Figure 6—figure supplement 1; Maes et al., 2001). Despite repeated attempts, we were not able to conjugate the plasmid into isolates outside of Clade I successfully. Each of the pFiD188Δ*att*-carrying isolates caused leafy galls on plants (Figure 6A). In addition, these isolates were no longer capable of causing increases in the growth of root hairs, unlike their corresponding near-isogenic genotypes (Figure 6B). Instead, the isolates carrying pFiD188Δ*att*, when compared to their near-isogenic plasmid-lacking genotypes, caused disease symptoms and significantly inhibited the growth of seedlings (Figure 6B–C; p-values were all <0.0001).

**Figure 6—figure supplement 1.**
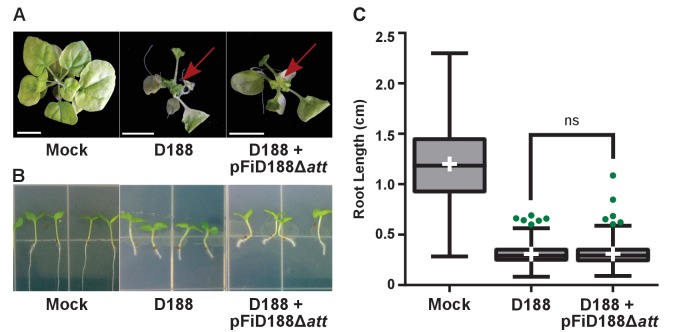
*Rhodococcus* isolate D188 with plasmid pFiD188Δ*att* is pathogenic. (**A**) Representative images of leafy galls on *N. benthamiana*. Images were taken 56 days post inoculation (dpi). Red arrow indicates leafy gall. (**B**) Representative images of root length of seedlings. Three-day-old *N. benthamiana* seedlings were inoculated with the indicated isolate of *Rhodococcus* or water (mock) and grown vertically for seven days under constant light. (**C**) Quantification of root lengths of *N. benthamiana* seedlings. Data for D188 +pFiD188Δ*att* are the same as those shown in Figure 6.

**Figure 6—figure supplement 2.**
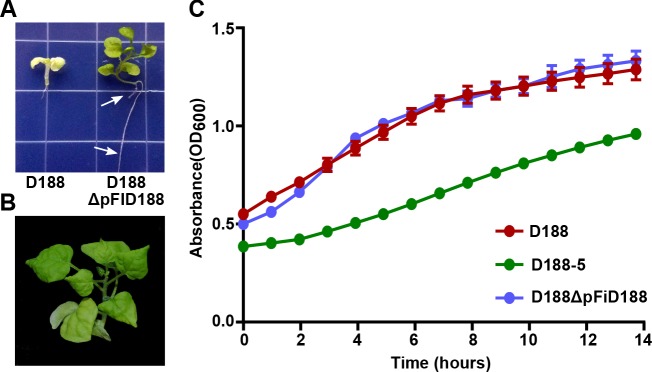
Eviction of the virulence plasmid reverts isolate D188 to a beneficial bacterium. (**A**) *Rhodococcus* D188ΔpFID188 causes changes in root architecture. White arrows indicate lateral roots and root hairs. The image was taken 42 days post inoculation (dpi); effects were seen earlier. (**B**) *Rhodococcus* D188ΔpFID188 is not pathogenic. The strain was inoculated onto *N. benthamiana*. The image was taken 56 dpi. (**C**) *Rhodococcus* isolate D188-5 is compromised in in vitro growth. *Rhodococcus* isolates D188, D188-5, and D188ΔpFiD188 were grown separately in LB media. Growth, determined using a Tecan plate reader, was quantified on the basis of OD_600_ readings for a period of 16 hr. One representative experiment of three is shown; all had similar results. Error bars indicate SEM. 10.7554/eLife.30925.019Figure 6—figure supplement 2—source data 1.Optical density values of culture-grown bacteria.

**Figure 6—figure supplement 3.**
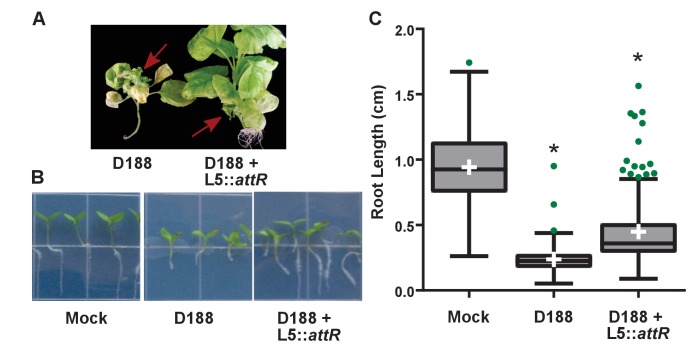
*Rhodococcus* isolate D188 carrying L5::*attR* is affected in virulence. (**A**) Representative images of leafy galls on *N. benthamiana*. Images were taken 56 days post inoculation (dpi). Red arrows indicate a leafy gall. (**B**) Representative images of the root lengths of seedlings. Three-day-old *N. benthamiana* seedlings were inoculated with the indicated isolate of *Rhodococcus* or water (mock) and grown vertically for seven days under constant light. (**C**) Quantification of root lengths of *N. benthamiana* seedlings. * indicates a significant difference compared to treatment with D188. 10.7554/eLife.30925.021Figure 6—figure supplement 3—source data 1.Lengths of *N. benthamiana* seedling roots 7 days after inoculation with isolate D188 +/- L5::*att*R.

The inverse transition was also demonstrated. We isolated a variant of D188, D188ΔpFiD188, which lacks the virulence plasmid. When inoculated onto roots of *N. benthamiana* seedlings, D188ΔpFiD188 caused changes to the architecture of root systems and no longer caused disease to mature plants (Figure 6B; Figure 6—figure supplement 2). We had to generate a new plasmid-lacking strain because the previously generated D188-5 is compromised in in vitro growth (Figure 1; Figure 6C; Figure 6—figure supplement 2; Desomer et al., 1988). Analysis of its genome sequence revealed a significant deletion of 25.4 kb from the chromosome (Supplementary file 1H). Most of the affected 25 genes have annotated functions implicated in housekeeping functions. Sequencing of D188ΔpFiD188 confirmed that it only lacked the virulence plasmid. This isolate also grew similarly to D188 and had no measurable fitness defects (Figure 6C; Figure 6—figure supplement 2).

**Figure 7.**
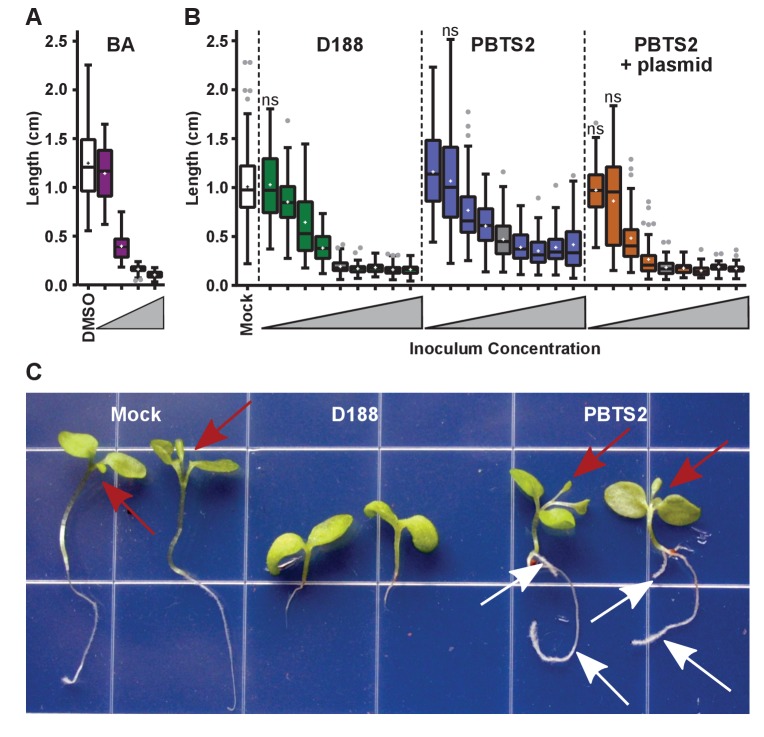
*Rhodococcus* has a dose-dependent effect on the root elongation of *N. benthamiana* seedlings. (**A**) Quantification of the seedling root lengths of plants grown in exogenously applied cytokinin (6-benyzlaminopurine; BA). Three-day-old *N. benthamiana* seedlings were transferred to media supplemented with BA (0.01, 0.1, 1.0, and 10 µM) or dimethyl sulfoxide (DMSO). (**B**) Quantification of the root lengths of seedlings inoculated with increasing doses of *Rhodococcus*. Three-day-old *N. benthamiana* seedlings were inoculated with isolates D188, PBTS2, or PBTS2 + pFiD188Δ*att*, with doses ranging from 2.5 × 10^2^ to 1.0 × 10^12^ colony-forming units (cfu). The sample shaded in gray highlights the inoculum of OD_600_ = 0.5 (1x = 2.5 × 10^10^ cfu) used in all other assays. Inocula below this decrease in 100-fold intervals. Inocula above increase at 2x, 4x, 10x, and 20x. All treatments are significantly different from mock unless otherwise noted with ‘ns’. (**C**) Representative image of morphological changes in seedlings. Seedlings inoculated with *Rhodococcus* D188 or PBTS2 (5 × 10^11^ cfu; 10x typical amount) or water (mock) were photographed. Red arrows indicate true leaves. White arrows indicate lateral roots and the proliferation of root hairs. 10.7554/eLife.30925.024Figure 7—source data 1.

Only three loci on pFiD188 have been implicated in virulence. We have not been able to repeat results showing that the deletion mutant of *att* is attenuated in virulence (Figure 6—figure supplement 1; Crespi et al., 1992; Maes et al., 2001). But when constitutively expressing *attR*, a homolog of the LysR transcriptional regulator necessary for *att* gene expression, D188 caused unusual leafy galls on *N. benthamiana* (Figure 6—figure supplement 3). Unlike normal galls that terminate primary growth, those caused by the *attR*-overexpressing strain regained meristematic activity. When inoculated onto roots, the symptoms were more variable, but nonetheless similar to those caused by D188. The effects were significantly different relative to those seen in mock-inoculated seedlings (p-value<0.0001) or in those inoculated with D188 (p-value<0.0001). The *fas* locus is predicted to be necessary for *Rhodococcus* to produce and secrete a mix of cytokinins (Pertry et al., 2009). Approximately 0.1 μM of the synthetic cytokinin 6-benzylaminopurine (BA) was equivalent to a starting inoculum of only ~2.5×10^3^ colony-forming units (cfu) of D188 (Figure 7A). However, regardless of the amount of BA in the medium, the exogenously applied cytokinins only inhibited root elongation and did not provoke the thickening of stems or arrest plant growth.

**Figure 7—figure supplement 1.**
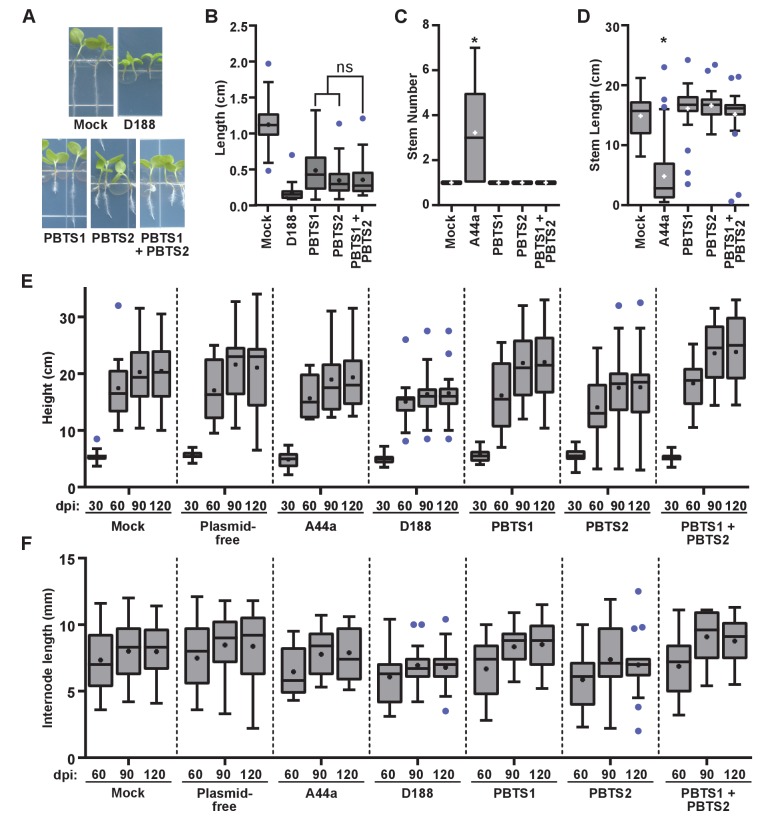
*Rhodococcus* isolates PBTS1 and PBTS2 do not cause disease symptoms in tested plant species. (**A**) Representative images of disease symptoms or beneficial effects on seedlings of *N. benthamiana*. Three-day-old seedlings were inoculated with *Rhodococcus* or water (mock) and grown vertically for seven days under constant light. (**B**) Quantification of seedling root length of *N. benthamiana*. All were significant relative to mock. There were no significant (ns) differences between treatments with PBTS1, PBTS2, or their mixture. (**C, D**) Quantification of stem number (**C**) and stem length (**D**) of peas inoculated with *Rhodococcus* or mock-inoculated. * indicates a significant difference when comparing to the mock treatment. (**E, F**) Quantification of height (**E**) and internode length (**F**) of pistachio UCB1 rootstock inoculated with *Rhodococcus* or mock-inoculated. No treatment effect was significantly different from the time-matched mock treatment. One representative experiment of three is shown. 10.7554/eLife.30925.026Figure 7—figure supplement 1—source data 1.Lengths of *N. benthamiana *seedling roots 7 days after growth in BA or inoculation with isolates D188 or PBTS2. 10.7554/eLife.30925.027Figure 7—figure supplement 1—source data 2.Number of stems of peas 14 days after inoculation with isolates D188, PBTS1, or PBTS2. 10.7554/eLife.30925.028Figure 7—figure supplement 1—source data 3.Length of stems of peas 14 days after inoculation with isolates D188, PBTS1, or PBTS2. 10.7554/eLife.30925.029Figure 7—figure supplement 1—source data 4.Heights of pistachio UCB1 210 days after inoculations with *Rhodococcus *isolates. 10.7554/eLife.30925.030Figure 7—figure supplement 1—source data 5.Internode lengths of pistachio UCB1 210 days after inoculations with *Rhodococcus *isolates.

### PBTS1 and PBTS2 are not outbreak strains

Our results show that PBTS1 and PBTS2 are not pathogenic on *N. benthamiana* (Figures 4 and 5). We next tested whether altering the dose influences the outcome of interaction between *N. benthamiana* and PBTS2. As inoculum levels of PBTS2 were increased, there was a greater reduction in root length (Figure 7B), but the effect was never to the same robustness and degree as that measured in seedlings infected with D188. In addition, PBTS2 did not cause thickening of stems or terminal arrest in the growth of the plant. At 28 days post-inoculation (dpi), the leaves of seedlings inoculated with the highest tested levels of PBTS2 had developed to the same stage as those of mock-inoculated seedlings, whereas D188-inoculated seedlings remained arrested in growth (Figure 7C). The roots of seedlings inoculated with PBTS2 also formed lateral roots. As inoculum levels of PBTS2 were decreased, there was less reduction of root length, and at the lowest dose tested, roots were significantly longer (1.161 cm; p-value = 0.0043) than those of mock-treated plants (1.007 cm; Figure 7B). A pathogenic PBTS2 strain carrying pFiD188Δ*att* showed a dose effect similar to that seen for D188 (Figure 7B; p-values were all >0.3584 for all within-dose comparisons).

To exclude the possibility that these results are due to incompatibility between PBTS1 and PBTS2 and *N. benthamiana*, other species of plants were tested. We used pea, an indicator species for confirming pathogenic *Rhodococcus*, and UCB-1 pistachio, reportedly the host of the epidemic. Both plant species failed to show disease symptoms, regardless of whether isolates were tested individually or in combination (Figure 7—figure supplement 1). Nine additional pistachio isolates that lack virulence genes also failed to cause disease. Four isolates, 14-687, 14-688, 14-694, and 14-700, were cultured from asymptomatic pistachio plants while five, SR18, AGD2M, AGD3B, AGD6D, and AGD6H, were cultured from symptomatic pistachio plants. Even pathogenic isolates D188 and A44a failed to cause disease symptoms in UCB-1 pistachio.

**Figure 8.**
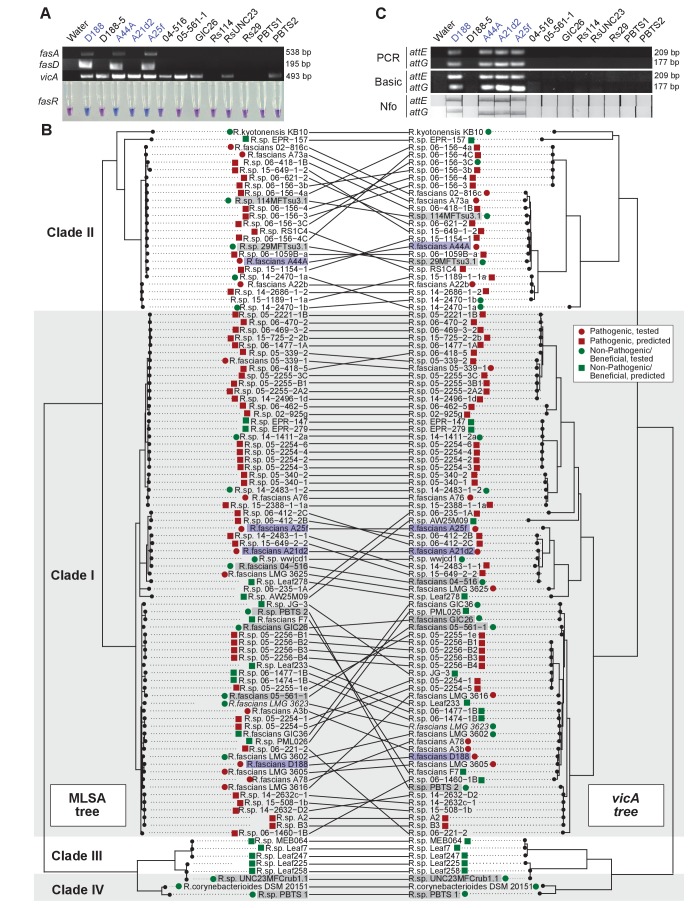
Use of the *vicA* gene does not discriminate pathogenic *Rhodococcus*. (**A**) (Top) Using locus-specific primers and DNA from the listed isolates, fragments of *fasA, fasD* and *vicA* were PCR amplified. Pathogenic isolates are labeled in blue; virulence-gene-lacking isolates are labeled in black. Products were resolved on a 1% TAE agarose gel. Amplicon sizes are listed to the right of the gel images. (Bottom) LAMP assay detection of *fasR* from the same DNA samples. A positive result is visualized by a blue color. Negative results are light purple. (**B**) Congruency between species (left) and *vicA* trees (right). Clades other than I–IV are not shown. Highlighted isolates are the same as in **A**, pathogenic isolates are highlighted in blue, virulence-gene-lacking isolates are highlighted in gray. (**C**) Standard endpoint PCR, RPA Basic, and RPA nfo were used to detect *attE* or *attG* from DNA extracted from isolates of *Rhodococcus*. Pathogenic isolates are labeled in blue. For PCR and RPA basic, product sizes are list to the right of the figure. For RPA nfo, the presence of the test band is indicative of a positive reaction; the control bands for all strips were confirmed (not shown).

Another aspect of the diagnosis of pistachio bushy top syndrome was the use of *vicA* to confirm pathogenic *Rhodococcus* (Stamler et al., 2015a, 2015b). Primers designed for *fasA* and *fasD* specifically amplified a product of expected size from pathogenic isolates D188, A44a, and A25f, and failed to amplify a product from any of the tested beneficial strains (Figure 8A; Supplementary file 1I; Nikolaeva et al., 2012; Serdani et al., 2013). The primers for *fasA*, and *fasD* failed to yield a product from A21d2 because this isolate carries an analog of the *fas* locus (Creason et al., 2014b). The molecular detection of *fasR* using a loop-mediated isothermal amplification (LAMP)-based assay specifically distinguished all tested pathogenic isolates from beneficial isolates.

The detection of *vicA* did not follow a pattern consistent with the pathogenicity phenotype (Figure 8A). It has a high false-positive rate and detected several, but not all, beneficial isolates. Our repeated attempts to amplify *vicA* from PBTS1 were unsuccessful; there are four and six mismatches between the two primers used for PCR and the *vicA* sequence from PBTS1. Homologs of *vicA* are predicted to be present in nearly all members of the Actinobacteria, including in all 407 *Rhodococcus* isolates for which genome sequences are available. The topologies of the *Rhodococcus* genus and the *vicA* trees are largely congruent, indicating that this locus is mostly vertically inherited, but with some evidence of recombination (Figure 8B).

To address the need for on-site molecular tools to distinguish pathogenic from beneficial genotypes, we used a new molecular detection method that is rapid, robust, and sensitive (Piepenburg et al., 2006). We targeted *attE* and *attG* because they are the most unique relative to all sequences in the databases and are conserved among the pathogenic isolates that we have examined (Creason et al., 2014b). The use of the primers for *attE* and *attG* in standard PCR and recombinase polymerase amplification (RPA) basic successfully amplified products of expected size from DNA of pathogenic strains, including A21d2 and A25f (Figure 8C). No products were detected when DNAs from beneficial strains were used as templates. An additional oligonucleotide probe that anneals within the amplified fragment was designed for RPA nfo, and when coupled with modified amplification primers, this probe was successful in detecting a product via lateral flow. This method was specific and discriminated between pathogenic and beneficial *Rhodococcus*. Moreover, RPA nfo can be completed in the absence of specialized equipment, and can yield results in just 30 min.

## Discussion

Whole-genome-enabled epidemiological studies have revealed local, global, and historical patterns for the transmission of human pathogens and have informed on health care (Comas et al., 2013; Croucher et al., 2011; Harris et al., 2010, 2013; Mutreja et al., 2011; Parkhill and Wren, 2011; Walker et al., 2013). Ours is a case study for using genomic epidemiology to uncover and explain the transmission patterns of phytopathogens in agricultural systems. The investigation of plasmid distribution highlighted the significant role of HGT in shaping the population structure of pathogenic bacteria and revealed challenges in modeling their transmission. Our analysis of chromosomal SNPs suggested that nurseries experience multiple and independent introductions of pathogenic *Rhodococcus*, exemplified by ‘a’ isolates observed in nurseries N15 and N8 (Figure 2). The link between isolates collected across time (‘b’) is indicative of a reservoir population that has a pathogenic genotype. The epidemiological links (‘c1’ and ‘c2’) of isolates from different nurseries support the possibility of point source outbreaks and suggest that the sources have reservoir populations.

**Figure 9.**
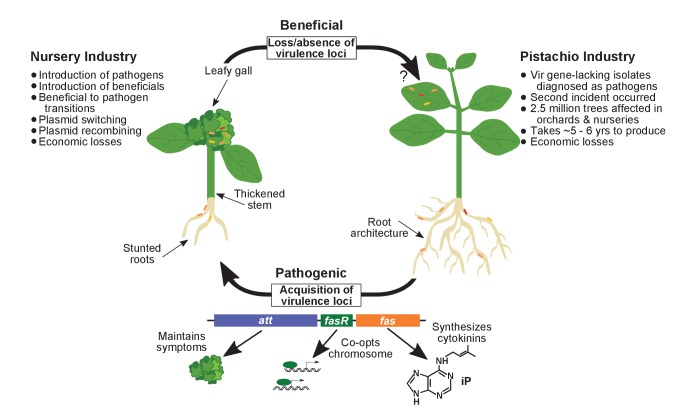
Model of the evolutionary transition in *Rhodococcus* and effects on agricultural sectors. The presence or absence of the virulence plasmid in eight species of *Rhodococcus*determines whether the bacterium is beneficial (right), and promotes growth in root architecture, or pathogenic (left), and causes leafy galls and inhibits primary growth. The virulence plasmid carries three loci identified as necessary for pathogenicity and their predicted functions are described. Horizontal gene transfer and evolutionary transitions affect *Rhodococcus* and impact the nursery industry (as indicated on the left of the figure). Virulence-gene-lacking isolates of *Rhodococcus* were diagnosed as outbreak strains and the probable misdiagnosis could have had detrimental impacts on the pistachio industry (as indicated on the right of the figure).

However, the distribution of plasmids also indicated that alternative processes may be occurring (Figures 3 and 9). First, two different plasmid types are associated with ‘c2’. This is not expected from an outbreak and is more consistent with different members of a lineage acquiring plasmids separately. Second, different plasmid variants are carried by isolates that are defined by genotypes associated with ‘a1’, ‘b’, and ‘c1’ (Figure 2). These are not expected patterns and probably reflect separate acquisitions of plasmids by different members of a lineage or the rapid and independent evolution of plasmids. Third, genetically distinct lineages of *Rhodococcus* at nursery N15 carry the same variant of plasmid. This is best explained by multiple lineages acquiring plasmids from a common donor population. Nurseries often produce a large variety of perennials and clonally propagated plants that are frequently handled and intensely managed in multiple production settings, and are often in regions that produce many different agricultural commodities. These are prime locations for different genotypes of *Rhodococcus* to interact and for plasmids to be transferred, switched, and evolved.

We also demonstrated that plants in agricultural systems are hosts to isolates of *Rhodococcus* that are probably beneficial associative symbionts (Supplementary file 1A; Vacheron et al., 2013). Root hairs are extensions that increase the surface area of roots, forming an extensive interface between plant and soil. Virulence-gene-lacking isolates of *Rhodococcus* caused significant increases in the number and length of root hairs, which may enable plants to be more efficient in the uptake of water and dissolved nutrients (Figure 5). This potentially beneficial growth-promoting phenotype is consistent with the finding of a number of reports identifying *Rhodococcus* within endophytic compartments and the rhizosphere of plants, and with the suggestions that the bacteria are enriched for by plants because of their beneficial traits (Bai et al., 2015; Bodenhausen et al., 2013; Bulgarelli et al., 2012; Francis et al., 2016; Hong et al., 2015, 2016; Lundberg et al., 2012; Qin et al., 2009, 2011; Salam et al., 2017). The associative symbionts are genetically diverse and represent 14 different species circumscribed by four sister clades (Figure 1; Supplementary file 1B). The mechanism by which isolates of *Rhodococcus* cause growth changes to plants is unknown, as genome mining efforts suggest that these traits are potentially novel (Figure 5—figure supplement 2).

Acquisition of a virulence plasmid by isolates representing eight species of Clades I and II is sufficient to drive an evolutionary transition (Figures 1 and 6). Loss of the plasmid reverted *Rhodococcus* to being beneficial, consistent with the hypothesis that virulence genes function irrespective of genomic background (Figure 6—figure supplement 2). In addition, we could not identify any chromosome-located genes that are enriched in pathogenic isolates, in comparison to non-pathogenic isolates, that could be potential candidate virulence genes. Evolutionary transitions, such as the switch from being a free-living, non-pathogenic lineage to being a pathogenic lineage, have been detected frequently (for example, by Bruto et al., 2017). The transition described for *Rhodococcus* is seamless and the mutualist to pathogen transition has been described only rarely (Sachs et al., 2011). It is possible that focus on characterizing binary outcomes in symbioses have obscured the true fluidity of symbioses. Similar transitions may have occurred in *Agrobacterium/Rhizobium*, a group of bacteria that express plasmid-mediated traits of significance to plant agriculture (de Lajudie et al., 1999; Glaeser et al., 2016; Hao et al., 2012; Lacroix and Citovsky, 2016; Wang et al., 2006).

Transitions and the rapid generation of new lineages of pathogens could occur in agricultural systems, where plants are frequently host to multiple *Rhodococcus* isolates of different genotypes. Pathogenic isolate 05-2254-6, which is associated with ‘a2’, is most similar to virulence-gene-lacking isolate, 14-1411-2a, also collected from N8. The 220 pairwise SNPs that differ between these two isolates exceeded the threshold used to define genotypes, but the two isolates are nonetheless closely related (Figures 1 and 2; Supplementary file 1C). This is not unique, as several pathogenic genotypes are related to virulence-gene-lacking genotypes, as indicated by their intermingling in the phylogeny and their connectivity in the network. The patterns involving distinct genotypes or plasmid types, such as ‘a1’ and ‘c2’, respectively, are also consistent with evolutionary transitions (Figures 2 and 3). An example that is similar to ‘a1’ is the genotype of D188 and LMG3605, both of which have different plasmid variants.

The limited genetic diversity in virulence plasmids within our dataset was unexpected and suggests that their common ancestor evolved recently (Figure 3—figure supplement 1). This is in dramatic contrast to the virulence plasmids of *Agrobacterium* species of bacteria, where the Ti and Ri plasmids form different types that can be easily distinguished on the basis of the phylogeny of a single, conserved virulence gene (Fuller et al., 2017). It is also remarkable that the virulence plasmid, which carries only three virulence loci, is sufficient for pathogenicity across eight genetically diverse species of *Rhodococcus* (Figures 1 and 9; Creason et al., 2014b; Francis et al., 2012; Letek et al., 2010). FasR is a predicted transcriptional regulator, and is probably key for reprogramming the genome to transition bacteria to pathogens. The roles of the other two loci are still unclear. Results presented here suggest that *att* contributes to the maintenance of disease symptoms, but the mechanism is unknown (Figure 6—figure supplement 3). The Fas-produced mix of cytokinins are predicted to be secreted into plants and necessary to cause disease symptoms, but the existing data are not consistent with this hypothesis (Pertry et al., 2009). Exceedingly low amounts of cytokinins are detected in culture-grown bacteria and plants have a variety of cytokinin-buffering mechanisms (Creason et al., 2014b; Kieber and Schaller, 2014; Pertry et al., 2009, 2010). A miniscule amount of starting bacterial inoculum is sufficient to provoke disease symptoms, and exogenous applications of cytokinins fail to phenocopy the effects of pathogenic *Rhodococcus* (Figure 7). Last, the cytokinin mixture model is challenged by the revelation that the fitness of D188-5, a key isolate used to develop the model, is severely compromised by a 25-kb deletion (Figure 6—figure supplement 2; Supplementary file 1H; Desomer et al., 1988; Maes et al., 2001; Pertry et al., 2009, 2010; Stes et al., 2011; Temmerman et al., 2001).

This study highlights the importance of understanding the genetic and phenotypic characteristics of an organism and the consequences of prematurely drawing conclusions from incomplete data. There is no evidence to suggest that HGT or evolutionary transitions confounded the diagnosis of pistachio, and we could not detect pathogenic *Rhodococcus* from symptomatic tissues of pistachio. We were unsuccessful in repeating results showing that PBTS1 and PBTS2, or other isolates cultured from pistachio, cause disease symptoms on plants (Figure 4; Figure 7—figure supplement 1). We could not amplify virulence genes from PBTS1 or PBTS2, but when a virulence plasmid was introduced into PBTS2, it was sufficient to transition PBTS2 to a pathogen of a plant species that is demonstrably susceptible to *Rhodococcus* (Figures 6 and 7B). Whether pistachio is even a host for pathogenic *Rhodococcus* is unresolved (Figure 7—figure supplement 1). Nevertheless, we recognize the insurmountable challenge in showing that there is no possibility that pathogenic *Rhodococcus* causes pistachio bushy top syndrome.

The results from this work prompted us to examine previous studies retrospectively (Figure 9; [Stamler et al., 2015a, 2015b]). There was a targeted search for *Rhodococcus*, the justification for which is unfounded because the symptoms on pistachio are unlike any produced by pathogenic *Rhodococcus* in any of the 100+ known hosts (Putnam and Miller, 2007). Bacteria were inexplicably cultured from asymptomatic leaves distal to symptomatic stem tissues. The key study did not include control strains or reproduce galling and graft failure, the most defining disease symptoms observed in field settings. A high inoculum of *Rhodococcus* was used and high doses of even beneficial bacteria can have costs (Figure 7). For example, some human diseases are caused by dysbiosis in which an imbalance of gut microbiotia causes disease (Bloom et al., 2011). In addition, hosts often employ mechanisms to regulate or ensure nonpersistent interactions with beneficial bacteria (Gutjahr and Parniske, 2013; Magori et al., 2009; Nyholm and McFall-Ngai, 2004; Reid et al., 2011; Wopereis et al., 2000).

Results based on molecular detection were similarly tenuous. The use of the *vicA* locus was misleading and led to conclusions regarding the pathogenicity of *Rhodococcus* (Figure 8). The reported amplification of fragments of *fasA* and *fasD* (GenBank accession numbers KP274062 and KP274064) from PBTS1 as well as *fasD* (KP274067) from PBTS2 cannot be reconciled with the absence of the genes from the genome sequences (Stamler et al., 2015b, 2016). We used a different technology to re-sequence independently prepared DNA from PBTS2 (Supplementary file 1F). The assembly is co-linear with the publically available sequence and lacks the virulence plasmid and virulence genes (Stamler et al., 2016). The possibility that virulence plasmids are unstable in populations grown outside of plant environments is not supported by the data. DNA extracted from bacteria grown in culture was used as a template in both PCR and whole-genome sequencing (Stamler et al., 2015b, 2016). We successfully sequenced plasmids from 64 culture-grown pathogenic isolates, which had undergone multiple transfers, and repeatedly and successfully detected pathogenic isolates cultured from symptomatic tissues (Creason et al., 2014b).

Some of the results of molecular detection were clearly artifacts. The two fragments reported to correspond to *fasD* are identical in sequence, each consisting of two short fragments of 147 and 194 nucleotides long that are artificially joined together by 280 ‘Ns’. In our dataset, A21d2 is the only sequenced pathogenic isolate that lacks *fasA*, but it also lacks a homolog of *fasD*. It is thus not expected that PBTS2 should have only *fasD* but no *fasA* (Stamler et al., 2015b). The most egregious artifact was the reported amplification of *vicA* from PBTS1 (GenBank accession number KP274063), which we could not reproduce (Figure 8). When the sequence of the reportedly amplified fragment was used as a query to BLAST search the PBTS1 genome sequence directly, we failed to identify a homologous region. When used in searches against publically available databases, the top hits other than KP274063 were NM-J PBTS (KR153287; 100% identity), DMS3-9 (KJ677035; 97% identity), D188 complete genome (CP015235; 96% identity), and PBTS2 complete genome (CP015220; 96% identity).

Another study posited that the virulence plasmids are unstable in populations persisting within plant environments (Nikolaeva et al., 2009). Genome sequences showed instead that *Rhodococcus* isolates are from genetically distinct lineages. This observation further emphasizes the need to use appropriate molecular diagnostic tools that discriminate pathogenic from non-pathogenic bacteria. LAMP to detect *fasR* or RPA nfo to detect *attE* or *attG* are both sensitive methods that have the additional benefit of being rapid and less dependent on specialized equipment (Figure 8; Serdani et al., 2013).

The data are consistent with the possibility that previous conclusions rest on a misdiagnosis of pistachio bushy top syndrome (Stamler et al., 2015a, 2015b). If so, *Rhodococcus* was implicated as pathogenic, irrespective of genotype and in disregard of the genetic and phenotypic diversity of the genus. This mindset conflates all bacteria as causative agents of disease and demotes the importance of bacteria in promoting the health of their hosts (Figure 9). This potential misdiagnosis of pistachio bushy top syndrome could be responsible for catastrophic effects. An estimated 2.5 million trees have been destroyed, resulting in tremendous economic loss. Efforts to identify the true cause were decelerated. Accusations regarding the source of *Rhodococcus* have introduced conflict into an industry struggling with a considerable and unfamiliar problem. Considering the data described here, previous conclusions should, at the very least, be tempered and recommendations for managing plants in which *Rhodococcus* bacteria have been detected, reexamined. Ideally, these findings will renew efforts to identify the true nature of the syndrome afflicting UCB-1 pistachio rootstocks.

Actinobacteria are prominent members of plant-associated communities, but the mechanisms and evolution of traits that are important for Gram-positive bacteria to reside in microbial communities and influence plant health are not well understood (Figure 9). Members of the *Rhodococcus* genus are excellent models for addressing this knowledge gap. A wealth of associated resources, such as an extensive and diverse collection of genotypes, associated genome sequences, and genetic tools have been developed for *Rhodococcus*. The members of this taxon can also interact with and benefit genetically tractable plant species. Last, the members of *Rhodococcus* are model organisms for characterizing evolutionary transitions between alternative symbiotic states.

## Materials and methods

### Bacterial isolates and growth conditions

*Rhodococcus* isolates used in this study are listed in Supplementary files 1A and F. Bacteria were maintained on solid LB medium at 28^o^C or grown overnight in LB medium at 28^o^C with shaking. Prior to conjugation, streptomycin-resistant *Rhodococcus* bacteria were selected for each of the recipient genotypes. Conjugations were done as previously described (Desomer et al., 1988). Donor and recipient strains were grown in yeast extract buffer (YEB) and shaken at 28°C. Each genotype was mixed at a ratio of 1:1 and filtered through a nitrocellulose filter (pore size, 0.45 μm; diameter, 25 mm; MilliporeSigma, Temecula, CA, USA). The filters were incubated on YEB agar plates for 24 to 28 h at 28°C. The cells were washed from the filter with 5 ml of a buffer containing 10 mM Tris-HCl pH 7.5 and 10 mM MgSO_4_ and then diluted and plated on YEB medium containing the appropriate antibiotic. *Escherichia coli* was grown on LB medium at 37^o^C. When appropriate, the medium was amended with 50 µg/ml of antibiotic kanamycin for *Rhodococcus* or *E. coli*. For growth curves, cultures of overnight-grown *Rhodococcus* were pelleted, washed, and resuspended at OD_600_ = 0.5 in a final volume of 200 µl of LB medium in 96-well flat-bottomed plates. The bacteria were grown for a period of 14 hr at 28^o^C with shaking in a Tecan Spark 10 m plate reader. Optical density (OD_600_) measurements were taken every hour. Three technical replicates were included for each isolate, and the experiment was repeated at least three times with similar results.

### Genome sequencing, assembly, and annotation

The Wizard genomic prep kit (Promega, Fitchburg, WI, USA) was used to extract genomic DNA from *Rhodococcus*. Directions for Gram-positive bacteria were followed. DNA was quantified with a Nanodrop spectrophotometer and adjusted to 50 ng/µl. Total genomic DNA was used to prepare Nextera XT libraries, and the resulting multiplexed libraries were sequenced on an Illumina HiSeq 3000 to generate 250mer paired end sequencing reads (Center for Genome Research and Biocomputing [CGRB], Oregon State University). Reads were processed as follows. FastQC was used to assess sequencing reads for quality (Andrews, 2014). BBduk v.35.82, with the parameters ‘ktrim = r k = 23 mink = 9 hdist = 1 minlength = 100 tpe tbo’, was used to remove adapter sequences (Bushnell, 2014). SPAdes v. 3.1.1, with the parameters ‘--careful -k 21,33,55,77,99’ was used to correct errors and to de novo assemble the reads into contigs (Bankevich et al., 2012). Blobtools was used to assess assemblies and guide elimination of contigs likely to be derived from contaminating bacteria (based on combined GC content, coverage, and contig annotation) (Kumar et al., 2013). Prokka was used to annotate the assembled genome sequences (Seemann, 2014).

### Phylogenetic analyses

Sequences for the maximum likelihood multi-locus sequence analysis (MLSA) tree were acquired using the autoMLSA tool (Davis Ii et al., 2016). The sequences for genes, *ftsY* (ABG98302.1), *infH* (ABG98417.1), *rpoB* (ABG93773.1), *rsmA* (ABG97450.1), *secY* (ABG97930.1), *tsaD* (ABG97962.1), and *ychF* (ABG97656.1) from the genome sequence of *Rhodococcus jostii* RHA1 were translated and used as queries in TBLASTN v. 2.2.31 searches against the assembled genome sequences and the NCBI nt database, masked to *Rhodococcus* (Adékambi et al., 2011; accessed 12/2016). Of those from NCBI nt, eight strains lacking all seven sequences and/or duplicate results were removed from the analysis. The sequences were aligned using MAFFT v. 6.864b with default settings (Katoh and Standley, 2013). A RAxML accessory script was used to determine the best-fitting protein model for each protein sequence alignment. Phylogenetic trees (100 ML searches, ‘autoMRE’ criterion bootstrap replicates) were generated using RAxML v. 8.1.17 with a partitioned alignment of the MLSA protein sequences (Stamatakis, 2014).

A similar analysis using *R. fascians* D188 *vicA* (AMY55488.1) as a query was used to acquire and assemble a phylogeny of 162 malate synthase gene sequences from the NCBI nr database masked to *Rhodococcus* (accessed 04/2017). A cophylo plot of the MLSA and *vicA* trees was generated using the R package phytools (Revell, 2012).

For the genes present in 95% of the virulence plasmids, sequences were concatenated using the R package EvobiR SuperMatrix function prior to constructing phylogenies (Blackmon and Adams, 2015).

Only bootstrap values greater than 50 are shown.

### Genome analyses and bioinformatics tools

Bowtie2 v. 2.2.3, with the option ‘--local’, was used to align reads to the chromosome references sequences of D188 (CP015235.1) or A44a (GCF_000760735.1), based on the clade assignment of the corresponding isolates (Langmead et al., 2009). Alignments were converted to bam format using samtools v. 0.1.18 and read groups were added using Picard tools v. 2.0.1 (Li et al., 2009; Picard Tools, 2015). GATK v. 3.7 HaplotypeCaller and the options ‘-ERC GVCF -ploidy 1’ were used to call variants for each isolate, and the data were then combined using GenotypeGVCFs (McKenna et al., 2010). Variants were filtered using the R package vcfR with depth filtering using quantile probabilities of 0.25 and 0.75 as cutoffs and a minimum of four reads, as well as a missing data cutoff of 20% (Knaus and Grünwald, 2017). Variants were converted into a fasta alignment using bcftools v. 1.3–14-ge0890a1 vcf-to-tab and the perl script vcftab-to-fasta (Li et al., 2009; Chen, 2012). Genotypes were called based on a threshold of 25 SNPs, and bitwise distances, using the R package poppr, were used to assemble minimum spanning networks (Kamvar et al., 2014).

Pairwise average nucleotide identity (ANI) between *Rhodococcus* isolates was calculated using autoANI (Davis Ii et al., 2016).

Get_homologues v. 20170418 with MCL clustering was used to cluster genes from 206 *Rhodococcus* genomes into orthologous groups (Contreras-Moreira and Vinuesa, 2013). The parse_pangenome_matrix.pl script of get_homologues was used to identify genes enriched (with a 95% threshold) in genomes of *Rhodococcus* in the four plant-associated clades.

To identify pathogenicity loci in sequenced isolates, *fasR*, *fas* and *att* were used as queries in TBLASTN searches against genome assemblies. CONTIGuator was used to map assembled contigs to the reference strain D188 to search for sequences corresponding to pFiD188 or pFID188-like plasmids (Galardini et al., 2011). Get_homologues v. 20170418 was used to cluster genes from each of the virulence plasmids as well as the other *Rhodococcus* linear plasmids (Francis et al., 2012). Plasmids were clustered on the basis of gene presence/absence using binary distances and Ward's method for clustering (ward.D2). dbCAN HMMs 5.0 was downloaded and used with *ad hoc* scripts to identify CAZYmes from translated genome sequences (Yin et al., 2012). The antiSMASH database was downloaded on 06/2017 and analyzed using antiSMASH ver. 4.0 (Blin et al., 2017). Queries used in TBLASTN searches were gene sequences ascertained from searching the literature (Bruto et al., 2014; Glick, 2012; Sparacino-Watkins et al., 2014).

HISAT2 was used to align sequencing reads from strain D188-5 to the D188 reference genome sequence. Variants (SNPs) were called using freebayes, filtered to those with quality score greater than 20 using vcffilter, and annotated using SNPdat v. 1.0.5 (Doran and Creevey, 2013).

### Plant growth conditions, assays, and data analysis

Seedling root inhibition assays were performed as described previously, with the exception that after bacteria were adjusted to OD_600_ = 0.5, they were sometimes diluted or concentrated (Creason et al., 2014b). At least 100 seedlings were assayed per treatment. Images were taken at 7 days post inoculation (dpi) and data were analyzed. For root hair quantification, a dissecting microscope, equipped with a camera, was used to capture images at 10 and 25 dpi. Root hairs within a 1 cm segment, 1 cm below the stem were quantified using ImageJ (Schneider et al., 2012). For cytokinin inhibition assays, three-day-old germinated seedlings were transplanted to MS (half-strength MS, 0.5M MES) medium containing DMSO (control) or 0.01–10.0 µM 6-benzylaminopurine (BA) and then grown and quantified in the same manner.

Leafy galls were induced using the decapitation method on four-week-old *N. benthamiana* plants (Creason et al., 2014b). Images were taken 28 dpi.

*Pisum sativum* ‘Alaska’ seeds were surface-sterilized in 70% ethanol for 1 min, 10% bleach for 10 min, and washed three times with sterile water. Seeds were soaked in sterile water for 60 min and then plated on water agar (15 g agar/L). Plates were incubated at 23°C until radicles were approximately 5 mm. Germinated seeds were soaked in suspension of *Rhodococcus* isolates (OD_600_ = 0.2) or 10 mM MgCl_2_ buffer for 45 min. Ten seeds per treatment were included in each experiment. Inoculated seeds were placed in sterile test tubes containing 5 ml of Hoagland’s nutrient agar. Samples were incubated for 14 days at 23°C with a 16/8 light/dark cycle. Stem number and length were quantified at 14 dpi.

Infection of UCB-1 pistachio was done, with minor modifications, according to previously described protocols (Stamler et al., 2015b). Briefly, control isolates, PBTS1, PBTS2, and a 1:1 mixture of PBTS1 and PBTS2 were suspended in 10 mM MgCl_2_ (final OD_600_ = 0.7). Treatment groups consisting of 15 seedlings were spray-inoculated with 200 ml of bacterial suspension. A mock-inoculated control group was sprayed with 200 ml of 10 mM MgCl_2_. Inoculated seedlings were placed in humidity chambers for 14 days and the plants were maintained in a greenhouse for seven months. Tree height and internode length were measured at 30 day intervals, and the final measurements were recorded at 210 dpi.

Unless indicated, all experiments were repeated at least three times with similar results. For all data sets, outliers were identified using the ROUT method (Q = 1%) and removed. Data were analyzed using One-way or Two-way ANOVA followed by Tukey’s multiple comparisons test (GraphPad Prism v.7, GraphPad Software, La Jolla, CA, USA). Box and whisker plots were generated using the Tukey method; colored dots indicate outliers. Means are indicated by +.

### Nucleic acid manipulations

The *attR* gene was PCR-amplified from D188 genomic DNA and subcloned downstream to the L5 bacteriophage promoter in vector pJDC165 (Jeff Cirillo, Texas A and M). The L5::*attR* construct was verified via Sanger sequencing. *Rhodococcus* competent cells were prepared from overnight-grown 3 ml cultures. Cells were pelleted and washed twice with sterile, cold dH_2_O, followed by one wash with sterile, cold 10% glycerol. Cells were resuspended in 50 µl 10% glycerol. Plasmid DNA (0.5–1 µg) was added to the cells. After 30 min of incubation on ice, the cells were electroporated in 1 mm gap cuvettes at 2.2 kV. Cells were resuspended in 250 µl SOC medium and incubated at 28°C with shaking for 16 hr prior to plating on LB medium with appropriate antibiotics.

For PCR, the following were used: 1x ThermoPol reaction buffer (New England Biolab, Ipswich, MA, USA), 200 μM dNTPs, 0.2 μM of each primer, 50 ng genomic DNA template, 0.625 units *Taq* DNA polymerase (New England Biolab, Ipswich, MA, USA), in a final volume of 25 μl. PCR conditions were 95°C, 3 min; 30 cycles of 95°C for 30 s, 55°C for 30 s, 72°C for 1 min; 72°C for 10 min; 16°C hold. Reactions with water, instead of a DNA template, were used as a negative control.

For LAMP, the reaction mixture was as follows: 0.5 ng DNA template, 1x ThermoPol reaction buffer (New England Biolab, Ipswich, MA, USA), 5 mM MgSO_4_, 140 µM dNTPs, 146 µM hydoxynaphthol blue (HNB), 1.6 µM each 16FIP and 16BIP primers, 0.2 µM each 16F3 and 16B3 primers, and 12 U Bst polymerase in a final volume of 25 µl. Reactions were incubated at 64°C for 60 min and then cooled to 4°C. Tubes were centrifuged briefly at 8000 rpm.

RPA was done per the manufacturer’s instructions (TwistAmp Basic, TwistDx Limited, Cambridge, UK). Reactions consist of 0.48 μM per primer, 29.5 μl rehydration buffer, 12.2 μl water, and 1.0 μl genomic DNA. A volume of 2.5 μl 280 mM magnesium acetate (MgAc) was added to initiate the reaction. The reaction was incubated at 37°C for 30 min (Fuller et al., 2017). Products were purified using the QIAquick PCR purification kit (Qiagen, Germany), run out on a 2.0% agarose gel, stained with ethidium bromide, and visualized under UV light. Products were verified via Sanger sequencing.

RPA reactions coupled to lateral flow detection were comprised of 0.42 μM forward primer, 0.42 μM biotin-labeled reverse primer, 0.12 μM probe, 29.5 μM rehydration buffer, 12.2 μl water, and 1.0 μl of 25 ng/μl genomic DNA. Reactions were added to a freeze-dried pellet provided by the manufacturer (TwistAmp nfo, TwistDx Limited, Cambridge, UK) with the subsequent addition of 2.5 μl of 280 mM MgAc to initiate the reaction. Following subsequent incubation at 37°C for 30 min, the dual-labelled amplicon was visualized using a lateral flow dipstick (Milenia Biotec GMbH, Germany). One microliter of the RPA product was diluted in 49 μl 1.0x PBST and 10 μl of the dilution were applied to the base of the dipstick, which was subsequently submerged in 100 μl 1.0 PBST at room temperature until the visualization of the positive control band, typically lasting two minutes.

Sequences of primers and probes, and their modifications, are described in Supplementary file 1I.

## Additional files

Supplementary file 1. Metadata and other supporting information. (A)Characteristics of *Rhodococcus* isolates sequenced in this study. (B) Average nucleotide identity of *Rhodococcus*. (C) Single nucleotide polymorphisms (SNPs) for Clade I isolates of *Rhodococcus*. (D) Single nucleotide polymorphisms (SNPs) for Clade II isolates of *Rhodococcus*. (E) List of 123 genes present in 95% or more plasmid sequences. (F) Characteristics of *Rhodococcus* isolates used in this study. (G) Genes enriched in Clade I–IV members of *Rhodococcus*. (H) Polymorphisms between *Rhodococcus* D188 and D188-5. (I) Sequences of primers and probes used in this study.

Transparent reporting form

### Major datasets

The following dataset was generated:

Elizabeth A SavorySkylar L FullerAlexandra J WeisbergWilliam J ThomasMichael I GordonDanielle M StevensAllison L CreasonMaryna SerdaniMichele S WisemanNiklaus J GrünwaldMelodie L PutnamJeff H Chang2017Sequencing of plant-associated Rhodococcushttps://www.ncbi.nlm.nih.gov/bioproject/PRJNA395383/Publicly available at NCBI BioProject (accession no: PRJNA395383)

The following previously published dataset was used:

A L CreasonO M VandeputteE A SavoryE W Davis IIet al2014Analysis of Genome Sequences from Plant Pathogenic Rhodococcus Reveals Genetic Novelties in Virulence Locihttps://www.ncbi.nlm.nih.gov/genome/?term=PRJNA233522Publicly available at NCBI Genome (accession no: PRJNA233522)

## References

[bib1] Adékambi T, Butler RW, Hanrahan F, Delcher AL, Drancourt M, Shinnick TM (2011). Core gene set as the basis of multilocus sequence analysis of the subclass Actinobacteridae. PLoS ONE.

[bib2] Andrews S (2014). http://www.bioinformatics.babraham.ac.uk/projects/fastqc.

[bib3] Bai Y, Müller DB, Srinivas G, Garrido-Oter R, Potthoff E, Rott M, Dombrowski N, Münch PC, Spaepen S, Remus-Emsermann M, Hüttel B, McHardy AC, Vorholt JA, Schulze-Lefert P (2015). Functional overlap of the Arabidopsis leaf and root microbiota. Nature.

[bib4] Bankevich A, Nurk S, Antipov D, Gurevich AA, Dvorkin M, Kulikov AS, Lesin VM, Nikolenko SI, Pham S, Prjibelski AD, Pyshkin AV, Sirotkin AV, Vyahhi N, Tesler G, Alekseyev MA, Pevzner PA (2012). SPAdes: a new genome assembly algorithm and its applications to single-cell sequencing. Journal of Computational Biology.

[bib5] Barea JM, Pozo MJ, Azcón R, Azcón-Aguilar C (2005). Microbial co-operation in the rhizosphere. Journal of Experimental Botany.

[bib6] Blackmon H, Adams R (2015). https://zenodo.org/record/30938#.Wh5IHdSF5xA.

[bib7] Blin K, Wolf T, Chevrette MG, Lu X, Schwalen CJ, Kautsar SA, Suarez Duran HG, de los Santos ELC, Kim HU, Nave M, Dickschat JS, Mitchell DA, Shelest E, Breitling R, Takano E, Lee SY, Weber T, Medema MH (2017). antiSMASH 4.0—improvements in chemistry prediction and gene cluster boundary identification. Nucleic Acids Research.

[bib8] Bloom SM, Bijanki VN, Nava GM, Sun L, Malvin NP, Donermeyer DL, Dunne WM, Allen PM, Stappenbeck TS (2011). Commensal *Bacteroides* species induce colitis in host-genotype-specific fashion in a mouse model of inflammatory bowel disease. Cell Host & Microbe.

[bib9] Bodenhausen N, Horton MW, Bergelson J (2013). Bacterial communities associated with the leaves and the roots of *Arabidopsis thaliana*. PLoS One.

[bib10] Bruto M, James A, Petton B, Labreuche Y, Chenivesse S, Alunno-Bruscia M, Polz MF, Le Roux F (2017). *Vibrio crassostreae*, a benign oyster colonizer turned into a pathogen after plasmid acquisition. The ISME Journal.

[bib11] Bruto M, Prigent-Combaret C, Muller D, Moënne-Loccoz Y (2014). Analysis of genes contributing to plant-beneficial functions in plant growth-promoting rhizobacteria and related proteobacteria. Scientific Reports.

[bib12] Bulgarelli D, Rott M, Schlaeppi K, Ver Loren van Themaat E, Ahmadinejad N, Assenza F, Rauf P, Huettel B, Reinhardt R, Schmelzer E, Peplies J, Gloeckner FO, Amann R, Eickhorst T, Schulze-Lefert P (2012). Revealing structure and assembly cues for Arabidopsis root-inhabiting bacterial microbiota. Nature.

[bib13] Bushnell B (2014). http://sourceforge.net/projects/bbmap/.

[bib14] Chen J (2012). https://github.com/JinfengChen/vcf-tab-to-fasta.

[bib15] Comas I, Coscolla M, Luo T, Borrell S, Holt KE, Kato-Maeda M, Parkhill J, Malla B, Berg S, Thwaites G, Yeboah-Manu D, Bothamley G, Mei J, Wei L, Bentley S, Harris SR, Niemann S, Diel R, Aseffa A, Gao Q, Young D, Gagneux S (2013). Out-of-Africa migration and Neolithic coexpansion of *Mycobacterium tuberculosis* with modern humans. Nature Genetics.

[bib16] Contreras-Moreira B, Vinuesa P (2013). GET_HOMOLOGUES, a versatile software package for scalable and robust microbial pangenome analysis. Applied and Environmental Microbiology.

[bib17] Creason AL, Davis EW, Putnam ML, Vandeputte OM, Chang JH (2014a). Use of whole genome sequences to develop a molecular phylogenetic framework for *Rhodococcus fascians* and the *Rhodococcus* genus. Frontiers in Plant Science.

[bib18] Creason AL, Vandeputte OM, Savory EA, Davis EW, Putnam ML, Hu E, Swader-Hines D, Mol A, Baucher M, Prinsen E, Zdanowska M, Givan SA, El Jaziri M, Loper JE, Mahmud T, Chang JH (2014b). Analysis of genome sequences from plant pathogenic *Rhodococcus* reveals genetic novelties in virulence loci. PLoS One.

[bib19] Crespi M, Messens E, Caplan AB, van Montagu M, Desomer J (1992). Fasciation induction by the phytopathogen *Rhodococcus fascians* depends upon a linear plasmid encoding a cytokinin synthase gene. The EMBO journal.

[bib20] Crespi M, Vereecke D, Temmerman W, Van Montagu M, Desomer J (1994). The *fas* operon of *Rhodococcus fascians* encodes new genes required for efficient fasciation of host plants. Journal of Bacteriology.

[bib21] Croucher NJ, Harris SR, Fraser C, Quail MA, Burton J, van der Linden M, McGee L, von Gottberg A, Song JH, Ko KS, Pichon B, Baker S, Parry CM, Lambertsen LM, Shahinas D, Pillai DR, Mitchell TJ, Dougan G, Tomasz A, Klugman KP, Parkhill J, Hanage WP, Bentley SD (2011). Rapid pneumococcal evolution in response to clinical interventions. Science.

[bib22] Davis Ii EW, Weisberg AJ, Tabima JF, Grunwald NJ, Chang JH (2016). Gall-ID: tools for genotyping gall-causing phytopathogenic bacteria. PeerJ.

[bib23] de Carvalho CC, Costa SS, Fernandes P, Couto I, Viveiros M (2014). Membrane transport systems and the biodegradation potential and pathogenicity of genus *Rhodococcus*. Frontiers in Physiology.

[bib24] de Lajudie P, Willems A, Nick G, Mohamed SH, Torck U, Coopman R, Filali-Maltouf A, Kersters K, Dreyfus B, Lindström K, Gillis M (1999). *Agrobacterium* bv. 1 strains isolated from nodules of tropical legumes. Systematic and Applied Microbiology.

[bib25] Desomer J, Dhaese P, Van Montagu M (1988). Conjugative transfer of cadmium resistance plasmids in *Rhodococcus fascians* strains. Journal of Bacteriology.

[bib26] Doran AG, Creevey CJ (2013). Snpdat: easy and rapid annotation of results from de novo snp discovery projects for model and non-model organisms. BMC Bioinformatics.

[bib27] Drogue B, Doré H, Borland S, Wisniewski-Dyé F, Prigent-Combaret C (2012). Which specificity in cooperation between phytostimulating rhizobacteria and plants?. Research in Microbiology.

[bib28] Francis I, De Keyser A, De Backer P, Simón-Mateo C, Kalkus J, Pertry I, Ardiles-Diaz W, De Rycke R, Vandeputte OM, El Jaziri M, Holsters M, Vereecke D (2012). pFiD188, the linear virulence plasmid of *Rhodococcus fascians* D188. Molecular Plant-Microbe Interactions.

[bib29] Francis IM, Stes E, Zhang Y, Rangel D, Audenaert K, Vereecke D (2016). Mining the genome of Rhodococcus fascians, a plant growth-promoting bacterium gone astray. New Biotechnology.

[bib30] Fuller SL, Savory EA, Weisberg AJ, Buser JZ, Gordon MI, Putnam ML, Chang JH (2017). Isothermal amplification and lateral-flow assay for detecting crown-gall-causing agrobacterium spp. Phytopathology.

[bib31] Galardini M, Biondi EG, Bazzicalupo M, Mengoni A (2011). CONTIGuator: a bacterial genomes finishing tool for structural insights on draft genomes. Source Code for Biology and Medicine.

[bib32] Glaeser SP, Imani J, Alabid I, Guo H, Kumar N, Kämpfer P, Hardt M, Blom J, Goesmann A, Rothballer M, Hartmann A, Kogel KH (2016). Non-pathogenic *Rhizobium radiobacter* F4 deploys plant beneficial activity independent of its host *Piriformospora indica*. The ISME Journal.

[bib33] Glick BR (2012). Plant growth-promoting bacteria: mechanisms and applications. Scientifica.

[bib34] Glick BR (2014). Bacteria with ACC deaminase can promote plant growth and help to feed the world. Microbiological Research.

[bib35] Gutjahr C, Parniske M (2013). Cell and developmental biology of arbuscular mycorrhiza symbiosis. Annual Review of Cell and Developmental Biology.

[bib36] Hao X, Xie P, Johnstone L, Miller SJ, Rensing C, Wei G (2012). Genome sequence and mutational analysis of plant-growth-promoting bacterium *Agrobacterium tumefaciens* CCNWGS0286 Isolated from a zinc-lead mine tailing. Applied and Environmental Microbiology.

[bib37] Harris SR, Cartwright EJ, Török ME, Holden MT, Brown NM, Ogilvy-Stuart AL, Ellington MJ, Quail MA, Bentley SD, Parkhill J, Peacock SJ (2013). Whole-genome sequencing for analysis of an outbreak of meticillin-resistant *Staphylococcus aureus*: a descriptive study. The Lancet Infectious Diseases.

[bib38] Harris SR, Feil EJ, Holden MT, Quail MA, Nickerson EK, Chantratita N, Gardete S, Tavares A, Day N, Lindsay JA, Edgeworth JD, de Lencastre H, Parkhill J, Peacock SJ, Bentley SD (2010). Evolution of MRSA during hospital transmission and intercontinental spread. Science.

[bib39] Hong CE, Jeong H, Jo SH, Jeong JC, Kwon SY, An D, Park JM (2016). A leaf-inhabiting endophytic bacterium, *rhodococcus* sp. kb6, enhances sweet potato resistance to black rot disease caused by *ceratocystis fimbriata*. Journal of Microbiology and Biotechnology.

[bib40] Hong CE, Jo SH, Moon JY, Lee J-S, Kwon S-Y, Park JM (2015). Isolation of novel leaf-inhabiting endophytic bacteria in *Arabidopsis thaliana* and their antagonistic effects on phytophathogens. Plant Biotechnology Reports.

[bib41] Kamvar ZN, Tabima JF, Grünwald NJ (2014). Poppr: an R package for genetic analysis of populations with clonal, partially clonal, and/or sexual reproduction. PeerJ.

[bib42] Katoh K, Standley DM (2013). MAFFT multiple sequence alignment software version 7: improvements in performance and usability. Molecular Biology and Evolution.

[bib43] Kieber JJ, Schaller GE (2014). Cytokinins. The Arabidopsis Book.

[bib44] Knaus BJ, Grünwald NJ (2017). vcfr: a package to manipulate and visualize variant call format data in R. Molecular Ecology Resources.

[bib45] Kumar S, Jones M, Koutsovoulos G, Clarke M, Blaxter M (2013). Blobology: exploring raw genome data for contaminants, symbionts and parasites using taxon-annotated GC-coverage plots. Frontiers in Genetics.

[bib46] Lacroix B, Citovsky V (2016). A functional bacterium-to-plant dna transfer machinery of *rhizobium etli*. PLOS Pathogens.

[bib47] Langmead B, Trapnell C, Pop M, Salzberg SL (2009). Ultrafast and memory-efficient alignment of short DNA sequences to the human genome. Genome Biology.

[bib48] Larkin MJ, Kulakov LA, Allen CC (2005). Biodegradation and Rhodococcus--masters of catabolic versatility. Current Opinion in Biotechnology.

[bib49] Lebeis SL, Paredes SH, Lundberg DS, Breakfield N, Gehring J, McDonald M, Malfatti S, Glavina del Rio T, Jones CD, Tringe SG, Dangl JL (2015). Plant microbiome. salicylic acid modulates colonization of the root microbiome by specific bacterial taxa. Science.

[bib50] Letek M, González P, Macarthur I, Rodríguez H, Freeman TC, Valero-Rello A, Blanco M, Buckley T, Cherevach I, Fahey R, Hapeshi A, Holdstock J, Leadon D, Navas J, Ocampo A, Quail MA, Sanders M, Scortti MM, Prescott JF, Fogarty U, Meijer WG, Parkhill J, Bentley SD, Vázquez-Boland JA (2010). The genome of a pathogenic rhodococcus: cooptive virulence underpinned by key gene acquisitions. PLoS Genetics.

[bib51] Li H, Handsaker B, Wysoker A, Fennell T, Ruan J, Homer N, Marth G, Abecasis G, Durbin R, 1000 Genome Project Data Processing Subgroup (2009). The sequence alignment/map format and SAMtools. Bioinformatics.

[bib52] Lin D, Koskella B (2015). Friend and foe: factors influencing the movement of the bacterium Helicobacter pylori along the parasitism-mutualism continuum. Evolutionary Applications.

[bib53] Lombard V, Golaconda Ramulu H, Drula E, Coutinho PM, Henrissat B (2014). The carbohydrate-active enzymes database (CAZy) in 2013. Nucleic Acids Research.

[bib54] Lundberg DS, Lebeis SL, Paredes SH, Yourstone S, Gehring J, Malfatti S, Tremblay J, Engelbrektson A, Kunin V, Del Rio TG, Edgar RC, Eickhorst T, Ley RE, Hugenholtz P, Tringe SG, Dangl JL (2012). Defining the core *Arabidopsis thaliana* root microbiome. Nature.

[bib55] Maes T, Vereecke D, Ritsema T, Cornelis K, Thu HN, Van Montagu M, Holsters M, Goethals K (2001). The *att* locus of *Rhodococcus fascians* strain D188 is essential for full virulence on tobacco through the production of an autoregulatory compound. Molecular Microbiology.

[bib56] Magori S, Oka-Kira E, Shibata S, Umehara Y, Kouchi H, Hase Y, Tanaka A, Sato S, Tabata S, Kawaguchi M (2009). Too much love, a root regulator associated with the long-distance control of nodulation in *Lotus japonicus*. Molecular Plant-Microbe Interactions.

[bib57] McKenna A, Hanna M, Banks E, Sivachenko A, Cibulskis K, Kernytsky A, Garimella K, Altshuler D, Gabriel S, Daly M, DePristo MA (2010). The genome analysis toolkit: a mapreduce framework for analyzing next-generation dna sequencing data. Genome Research.

[bib58] Miteva VI, Sheridan PP, Brenchley JE (2004). Phylogenetic and physiological diversity of microorganisms isolated from a deep greenland glacier ice core. Applied and Environmental Microbiology.

[bib59] Mutreja A, Kim DW, Thomson NR, Connor TR, Lee JH, Kariuki S, Croucher NJ, Choi SY, Harris SR, Lebens M, Niyogi SK, Kim EJ, Ramamurthy T, Chun J, Wood JL, Clemens JD, Czerkinsky C, Nair GB, Holmgren J, Parkhill J, Dougan G (2011). Evidence for several waves of global transmission in the seventh cholera pandemic. Nature.

[bib60] Nikolaeva EV, Kang S, Olson TN, Kim S (2012). Real-time PCR detection of *rhodococcus fascians* and discovery of new plants associated with *r. fascians* in pennsylvania. Plant Health Progress.

[bib61] Nikolaeva EV, Park S-Y, Kang S, Olson TN, Kim SH (2009). Ratios of cells with and without virulence genes in *rhodococcus fascians* populations correlate with degrees of symptom development. Plant Disease.

[bib62] Nyholm SV, McFall-Ngai MJ (2004). The winnowing: establishing the squid-vibrio symbiosis. Nature Reviews Microbiology.

[bib63] Parkhill J, Wren BW (2011). Bacterial epidemiology and biology--lessons from genome sequencing. Genome Biology.

[bib64] Pertry I, Václavíková K, Depuydt S, Galuszka P, Spíchal L, Temmerman W, Stes E, Schmülling T, Kakimoto T, Van Montagu MC, Strnad M, Holsters M, Tarkowski P, Vereecke D (2009). Identification of *Rhodococcus fascians* cytokinins and their modus operandi to reshape the plant. PNAS.

[bib65] Pertry I, Václavíková K, Gemrotová M, Spíchal L, Galuszka P, Depuydt S, Temmerman W, Stes E, De Keyser A, Riefler M, Biondi S, Novák O, Schmülling T, Strnad M, Tarkowski P, Holsters M, Vereecke D (2010). *Rhodococcus fascians* impacts plant development through the dynamic *fas*-mediated production of a cytokinin mix. Molecular Plant-Microbe Interactions.

[bib66] Picard Tools (2015). http://broadinstitute.github.io/picard.

[bib67] Piepenburg O, Williams CH, Stemple DL, Armes NA (2006). DNA detection using recombination proteins. PLoS Biology.

[bib68] Pieterse CM, Zamioudis C, Berendsen RL, Weller DM, Van Wees SC, Bakker PA (2014). Induced systemic resistance by beneficial microbes. Annual Review of Phytopathology.

[bib69] Putnam ML, Miller ML (2007). *Rhodococcus fascians* in Herbaceous Perennials. Plant Disease.

[bib70] Qin S, Li J, Chen HH, Zhao GZ, Zhu WY, Jiang CL, Xu LH, Li WJ (2009). Isolation, diversity, and antimicrobial activity of rare actinobacteria from medicinal plants of tropical rain forests in Xishuangbanna, China. Applied and Environmental Microbiology.

[bib71] Qin S, Xing K, Jiang JH, Xu LH, Li WJ (2011). Biodiversity, bioactive natural products and biotechnological potential of plant-associated endophytic actinobacteria. Applied Microbiology and Biotechnology.

[bib72] Reid DE, Ferguson BJ, Hayashi S, Lin YH, Gresshoff PM (2011). Molecular mechanisms controlling legume autoregulation of nodulation. Annals of Botany.

[bib73] Revell LJ (2012). Phytools: an R package for phylogenetic comparative biology (and other things). Methods in Ecology and Evolution.

[bib74] Sachs JL, Skophammer RG, Regus JU (2011). Evolutionary transitions in bacterial symbiosis. PNAS.

[bib75] Salam N, Khieu TN, Liu MJ, Vu TT, Chu-Ky S, Quach NT, Phi QT, Narsing Rao MP, Fontana A, Sarter S, Li WJ (2017). Endophytic actinobacteria associated with *dracaena cochinchinensis* lour.: Isolation, diversity, and their cytotoxic activities. BioMed Research International.

[bib76] Schneider CA, Rasband WS, Eliceiri KW (2012). NIH Image to ImageJ: 25 years of image analysis. Nature Methods.

[bib77] Seemann T (2014). Prokka: rapid prokaryotic genome annotation. Bioinformatics.

[bib78] Serdani M, Curtis M, Miller ML, Kraus J, Putnam ML (2013). Loop-mediated isothermal amplification and polymerase chain reaction methods for specific and rapid detection of *Rhodococcus fascians*. Plant Disease.

[bib79] Soucy SM, Huang J, Gogarten JP (2015). Horizontal gene transfer: building the web of life. Nature Reviews Genetics.

[bib80] Spaepen S, Vanderleyden J, Remans R (2007). Indole-3-acetic acid in microbial and microorganism-plant signaling. FEMS Microbiology Reviews.

[bib81] Sparacino-Watkins C, Stolz JF, Basu P (2014). Nitrate and periplasmic nitrate reductases. Chem. Soc. Rev..

[bib82] Stamatakis A (2014). RAxML version 8: a tool for phylogenetic analysis and post-analysis of large phylogenies. Bioinformatics.

[bib83] Stamler RA, Heerema R, Randall JJ (2015a). First Report of Phytopathogenic *Rhodococcus* Isolates on Pistachio Bushy Top Syndrome ‘UCB-1’ Rootstock in New Mexico. Plant Disease.

[bib84] Stamler RA, Kilcrease J, Kallsen C, Fichtner EJ, Cooke P, Heerema RJ, Randall JJ (2015b). First Report of *Rhodococcus* Isolates Causing Pistachio Bushy Top Syndrome on ‘UCB-1’ Rootstock in California and Arizona. Plant Disease.

[bib85] Stamler RA, Vereecke D, Zhang Y, Schilkey F, Devitt N, Randall JJ (2016). Complete genome and plasmid sequences for *Rhodococcus fascians* D188 and draft sequences for *Rhodococcus* Isolates PBTS 1 and PBTS 2. Genome Announcements.

[bib86] Stes E, Vandeputte OM, El Jaziri M, Holsters M, Vereecke D (2011). A successful bacterial coup d'état: how *Rhodococcus fascians* redirects plant development. Annual Review of Phytopathology.

[bib87] Temmerman W, Ritsema T, Simón-Mateo C, Van Montagu M, Mironov V, Inzé D, Goethals K, Holsters M (2001). The *fas* locus of the phytopathogen *Rhodococcus fascians* affects mitosis of tobacco BY-2 cells. FEBS Letters.

[bib88] Temmerman W, Vereecke D, Dreesen R, Van Montagu M, Holsters M, Goethals K (2000). Leafy gall formation is controlled by *fasR*, an AraC-type regulatory gene in *Rhodococcus fascians*. Journal of Bacteriology.

[bib89] Vacheron J, Desbrosses G, Bouffaud ML, Touraine B, Moënne-Loccoz Y, Muller D, Legendre L, Wisniewski-Dyé F, Prigent-Combaret C (2013). Plant growth-promoting rhizobacteria and root system functioning. Frontiers in Plant Science.

[bib90] Verbon EH, Liberman LM (2016). Beneficial microbes affect endogenous mechanisms controlling root development. Trends in Plant Science.

[bib91] Vereecke D, Cornelis K, Temmerman W, Jaziri M, Van Montagu M, Holsters M, Goethals K (2002). Chromosomal locus that affects pathogenicity of *Rhodococcus fascians*. Journal of Bacteriology.

[bib92] Walker TM, Ip CL, Harrell RH, Evans JT, Kapatai G, Dedicoat MJ, Eyre DW, Wilson DJ, Hawkey PM, Crook DW, Parkhill J, Harris D, Walker AS, Bowden R, Monk P, Smith EG, Peto TE (2013). Whole-genome sequencing to delineate *Mycobacterium tuberculosis* outbreaks: a retrospective observational study. The Lancet Infectious Diseases.

[bib93] Wang LL, Wang ET, Liu J, Li Y, Chen WX (2006). Endophytic occupation of root nodules and roots of *Melilotus dentatus* by *Agrobacterium tumefaciens*. Microbial Ecology.

[bib94] Wopereis J, Pajuelo E, Dazzo FB, Jiang Q, Gresshoff PM, De Bruijn FJ, Stougaard J, Szczyglowski K (2000). Short root mutant of *Lotus japonicus* with a dramatically altered symbiotic phenotype. The Plant Journal.

[bib95] Yin Y, Mao X, Yang J, Chen X, Mao F, Xu Y (2012). dbCAN: a web resource for automated carbohydrate-active enzyme annotation. Nucleic Acids Research.

