# Correction: Evolutionary transitions between beneficial and phytopathogenic Rhodococcus challenge disease management

**DOI:** 10.7554/eLife.36350

**Published:** 2018-03-13

**Authors:** Elizabeth A Savory, Skylar L Fuller, Alexandra J Weisberg, William J Thomas, Michael I Gordon, Danielle M Stevens, Allison L Creason, Michael S Belcher, Maryna Serdani, Michele S Wiseman, Niklaus J Grünwald, Melodie L Putnam, Jeff H Chang

Savory EA, Fuller SL, Weisberg AJ, Thomas WJ, Gordon MI, Stevens DM, Creason AL, Belcher MS, Serdani M, Wiseman MS, Grünwald NJ, Putnam ML, Chang JH. 2017. Evolutionary transitions between beneficial and phytopathogenic *Rhodococcus* challenge disease management. *eLife* **6**:e30925. doi: 10.7554/eLife.30925.

Published 12, December 2017

Since the publication of this paper it has been brought to our attention that some details are missing from the paper. We have therefore corrected the paper as follows.

First, in the "Bacterial isolates and growth conditions" section of Materials and methods, we have added more details on the conjugation method by replacing the sentence “*Rhodococcus* conjugation were done as previously described (Desomer et al., 1988)" with the following sentence: “Prior to conjugation, streptomycin-resistant *Rhodococcus* bacteria were selected for each of the recipient genotypes. Conjugations were done as previously described (Desomer et al., 1988). Donor and recipient strains were grown in yeast extract buffer (YEB) and shaken at 28°C. Each genotype was mixed at a ratio of 1:1 and filtered through a nitrocellulose filter (pore size, 0.45 μm; diameter, 25 mm; MilliporeSigma, Temecula, CA, USA). The filters were incubated on YEB agar plates for 24 to 28 h at 28°C. The cells were washed from the filter with 5 ml of a buffer containing 10 mM Tris-HCl pH 7.5 and 10 mM MgSO_4_ and then diluted and plated on YEB medium containing the appropriate antibiotic.”

Second, Figure 5—figure supplement 1 did not include an image of a plant treated with the water control. An image of a plant treated with the water control has now been added to this figure, and the figure caption has been revised to read as follows:

**Figure 5—figure supplement 1.**
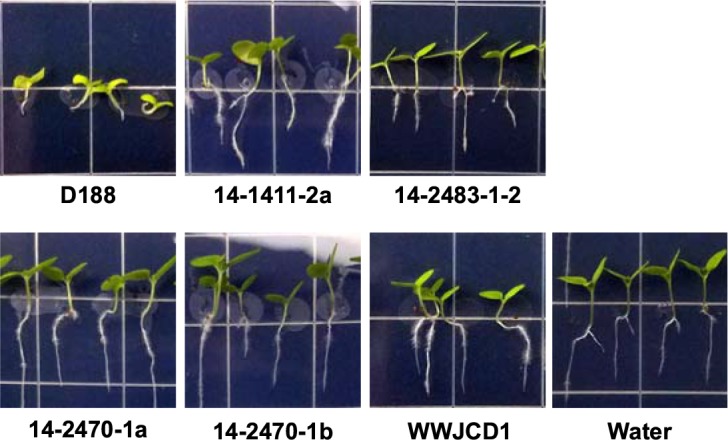
Five additional virulence gene-lacking isolates of *Rhodococcus* cause changes to root architecture. Representative images of seedlings inoculated with the indicated isolates of *Rhodococcus* or water (mock). The root lengths of seedlings inoculated with isolates lacking virulence genes were different from those of seedlings inoculated with isolate D188.

Third, we did not cite the work by Francis et al. (2016). We omitted this publication, because it did not provide any information on the evolutionary transition or demonstrate quantitative changes in root morphology. Also, in this previous study, an undescribed volume of a high concentration of bacteria was used. Last, we had discovered that the strain used by Francis et al. has a fitness cost and a 25 kb deletion in the genome. However, in retrospect we should have cited this prior work. This work has now been added to the list of references, and is cited at the end of the following sentence: “This potentially beneficial growth-promoting phenotype is consistent with the finding of a number of reports identifying *Rhodococcus* within endophytic compartments and the rhizosphere of plants, and with the suggestions that the bacteria are enriched for by plants because of their beneficial traits (Bai et al., 2015; Bodenhausen et al., 2013; Bulgarelli et al., 2012; Francis et al., 2016; Hong et al., 2015; 2016; Lundberg et al., 2012; Qin et al., 2009; 2011; Salam et al., 2017).”

Fourth, in the "PBTS1 and PBTS2 are not outbreak strains" section of Results, we did not report the origin of the nine other isolates that were cultured from pistachio and tested. The following sentence has been added to the article (after the sentence that begins “Nine additional pistachio isolates... ”): “Four isolates, 14-687, 14-688, 14-694, and 14-700, were cultured from asymptomatic pistachio plants while five, SR18, AGD2M, AGD3B, AGD6D, and AGD6H, were cultured from symptomatic pistachio plants.” The first four isolates were provided to us by a nursery while the latter five isolates were provided to us by Dr. Randall (see below). These nine isolates, as well as all other isolates reported in the article, are available upon request.

Fifth, we regrettably neglected to acknowledge all the individuals who provided strains. The first two sentences in the acknowledgements have been changed to read as follows: “This work would not have been possible without the contribution of plants from multiple nurseries, for which we are appreciative. We thank Dr. Edward Davis II for assistance with computational methods, Heidi Lederhos for her assistance in the laboratory, Dr. Zhian Kamvar for assistance with using poppr, Dr. Jeffrey Anderson (Oregon State University) for permitting use of the plate reader, Dr. Jeffery Dangl (University of North Carolina) for providing *Rhodococcus* spp. isolates 114MFTsu3.1, UNC23MFCrub1.1 and 29MFTsu3.1, Dr. Jeff Cirillo (Texas A & M) for pJDC165, Dr. Jennifer Randall (New Mexico State University) for *Rhodococcus* spp. isolates PBTS1, PBTS2, SR18, AGD2M, AGD3B, AGD6D, and AGD6H, Dr. Olivier Vandeputte for D188 carrying pFiD188Δ*att*, and staff in the Center for Genome Research and Biocomputing at Oregon State University, for sequencing services.”

We thank Jennifer Randall, her colleagues, and Danny Vereecke for drawing these matters to our attention.
