# Supplementary material for: Evolutionary transitions between beneficial and phytopathogenic Rhodococcus challenge disease management
Source: eLife. 2017 Dec 12;6:e30925. doi: 10.7554/eLife.30925 (PMC5726852; doi:10.7554/eLife.30925)
Supplement: Transparent reporting form [file elife-30925-transrepform.pdf]

eLIFE
1st Floor
24 Hills Road
Cambridge CB2 1JP, UK
P 01223 855340
W elifesciences.org
T @elife

eLife's transparent reporting form

We encourage authors to provide detailed information *within their submission* to facilitate the interpretation and replication of experiments. Authors can upload supporting documentation to indicate the use of appropriate reporting guidelines for health-related research (see EQUATOR Network), life science research (see the BioSharing Information Resource), or the ARRIVE guidelines for reporting work involving animal research. Where applicable, authors should refer to any relevant reporting standards documents in this form.

If you have any questions, please consult our Journal Policies and/or contact us: editorial@elifesciences.org.

Sample-size estimation

- You should state whether an appropriate sample size was computed when the study was being designed
- You should state the statistical method of sample size computation and any required assumptions
- If no explicit power analysis was used, you should describe how you decided what sample (replicate) size (number) to use

Please outline where this information can be found within the submission (e.g., sections or figure legends), or explain why this information doesn't apply to your submission:

No explicit power analysis was used to estimate sample size. In studies, other than those involving the manual enumeration of root hair number and root hair length, at least 50 seedlings were tested for each treatment. At least three independent biological replicates were performed. Information for individual experiments is described in the Materials and Methods section.

Replicates

- You should report how often each experiment was performed
- You should include a definition of biological versus technical replication
- The data obtained should be provided and sufficient information should be provided to indicate the number of independent biological and/or technical replicates
- If you encountered any outliers, you should describe how these were handled
- Criteria for exclusion/inclusion of data should be clearly stated
- High-throughput sequence data should be uploaded before submission, with a private link for reviewers provided (these are available from both GEO and ArrayExpress)

Please outline where this information can be found within the submission (e.g., sections or figure legends), or explain why this information doesn't apply to your submission:

eLife Sciences Publications, Ltd is a limited liability non-profit non-stock corporation incorporated in the State of Delaware, USA, with company number 5030732, and is registered in the UK with company number FC030576 and branch number BR015634 at the address 1st Floor, 24 Hills Road, Cambridge CB2 1JP | August 2014

The information concerning replicates can be found in the Materials and Methods section. We define a biological replicate as an experiment performed independently with regards to time, using biologically distinct samples that in our case were different plants and different cultures of bacteria. Biological replicates were repeated at least three times for bacterial growth curves and all plant assays. Within these independent biological replicates, numbers of technical replicates, which we define as the number of measurements of a parameter collected within a biological replicate, varied. These are described in the Materials and Methods section pertaining to each experiment. Outliers were identified using the ROUT method (Q = 1%) and removed.

Genomic sequence data has been uploaded to NCBI under BioProject# PRJNA395383. Each isolate has an assigned BioSample #, which are listed in Supplemental Table 1. Reviewers can access the data via NCBI.

Statistical reporting

- Statistical analysis methods should be described and justified
- Raw data should be presented in figures whenever informative to do so (typically when N per group is less than 10)
- For each experiment, you should identify the statistical tests used, exact values of N, definitions of center, methods of multiple test correction, and dispersion and precision measures (e.g., mean, median, SD, SEM, confidence intervals; and, for the major substantive results, a measure of effect size (e.g., Pearson's r, Cohen's d)
- Report exact p-values wherever possible alongside the summary statistics and 95% confidence intervals. These should be reported for all key questions and not only when the p-value is less than 0.05.

Please outline where this information can be found within the submission (e.g., sections or figure legends), or explain why this information doesn't apply to your submission:

Statistical analysis methods are described within the Materials and Methods section. Unless otherwise stated, all experiments were analyzed similarly. One-way or Two-way ANOVA followed by Tukey's multiple comparison test was done using GraphPad Prism v.7 (GraphPad Software, La Jolla California USA).

Analyses, including output from ANOVA and multiple comparison tests for all statistical tests, are provided in the table Statistical_Tests_for_Reviewers.xls. Each sheet within the file refers to the figure in which the data is shown.

All phylogenetic analyses were performed using RAxML. One hundred maximum likelihood (ML) tree searches were performed for each dataset, with the best-likelihood tree selected. The "autoMRE" criterion was used to determine the minimum number of bootstrap support trees to calculate, to provide bootstrap support on branches of the best tree.

(For large datasets, or papers with a very large number of statistical tests, you may upload a single table file with tests, Ns, etc., with reference to sections in the manuscript.)

Group allocation

eLife Sciences Publications, Ltd is a limited liability non-profit non-stock corporation incorporated in the State of Delaware, USA, with company number 5030732, and is registered in the UK with company number FC030576 and branch number BR015634 at the address 1st Floor, 24 Hills Road, Cambridge CB2 1JP | August 2014

- Indicate how samples were allocated into experimental groups (in the case of clinical studies, please specify allocation to treatment method); if randomization was used, please also state if restricted randomization was applied
- Indicate if masking was used during group allocation, data collection and/or data analysis

Please outline where this information can be found within the submission (e.g., sections or figure legends), or explain why this information doesn't apply to your submission:

This information does not apply to our submission.

Additional data files ("source data")

- We encourage you to upload relevant additional data files, such as numerical data that are represented as a graph in a figure, or as a summary table
- Where provided, these should be in the most useful format, and they can be uploaded as "Source data" files linked to a main figure or table
- Include model definition files including the full list of parameters used
- Include code used for data analysis (e.g., R, MatLab)
- Avoid stating that data files are "available upon request"

Please indicate the figures or tables for which source data files have been provided:

These are included as Supplemental Tables, which relate bacterial isolates to genome information and phenotypic traits. Because of the nature of the data, some information had to be codified to preserve anonymity. One table shows all pairwise ANI values. Two tables provide single nucleotide polymorphism calls.
